# Conceptualizing university students’ food choices based on theory of planned behavior

**DOI:** 10.12688/f1000research.123325.2

**Published:** 2024-10-21

**Authors:** Kshama Vishwakarma, Varalakshmi Chandra Sekaran, Vidya Patwardhan, Asha Kamath

**Affiliations:** 1 Associate Professor, Masters in Personal Management, DHMCT, Welcomgroup Graduate School of Hotel Administration, Manipal Academy of Higher Education, Manipal, Karnataka, 576104, India; 2 Assistant Professor, Global Public Health Policy and Governance, Prasanna School of Public Health, Manipal Academy of Higher Education, Manipal, Karnataka, 576104, India; 3 Professor & Coordinator, Centre for Hospitality and Tourism Research, Welcomgroup Graduate School of Hotel Administration, Manipal academy of Higher Education, Manipal, Karnataka, 576104, India; 4 Department of Applied Statistics and Data Science, Prasanna School of Public Health, P Manipal Academy of Higher Education, Manipal, Karnataka, 576104, India

**Keywords:** Food choices, university students, campus, university dining facility (UDF), qualitative research, healthy food, theory of planned behavior, food transition.

## Abstract

**Background:**

Several students use hostel facilities offered by their colleges and universities while pursuing higher education. A crucial change is witnessed in their food habits during their stay at the campus, which is vastly different from their home experience.

**Method:**

The study employs the theory of planned behavior to conceptualize and understand the food choices of university students while following the qualitative research methodology and the phenomenological approach. The snowball sampling method is applied to select participants for the study. We selected twenty-six undergraduate and postgraduate students pursuing programs in technical and health science from a private university at Udupi with residence in the university campus for two to four years. Interviews with candidates were conducted online and audio-recorded with participants’ consent.

**Results:**

The transcribed interviews were coded and categorized to obtain themes, which were then conceptualized to develop the model based on the theory of planned behaviour. The duration of their stay on campus allowed students to gain a perspective on the food events and the food they consumed at the university dining facility (UDF) guided by factors, such as taste, price, availability of time(during academic activity), accessibility to healthy food, academic stress and lack of knowledge on nutrition. Two factors emerged from the interviews, one, there was no display in the dining facility about the nutritional value of the food served; and, second, students preferred to dine out on unhealthy food rather than at the university because food from outside the campus, even though unhealthy, was easier on the pocket.

**Conclusion:**

The study provides insights into the role of UDF in serving healthy and nutritional food to students for better health and improved academic performance. This work brings to light the relationship between students’ food choices and their impact on academic performance.

## Introduction

Students pursuing higher education often avail hostel facilities offered by their colleges and universities. While residing in a new environment, students experience a notable shift in their lifestyle, diverging from familiar habits they maintain back home. ^4^ The shift to university life frequently includes the formation of new friendships, exposure to novel experiences, and personal development. Crucially, this transition aligns with a crucial phase for establishing enduring habits (Maillet et al, 2023). During this period crucial change is observed in their dietary preferences. ^4^ The dietary beliefs and actions of a person throughout their university years can significantly shape their habits in adulthood, potentially impacting the likelihood of developing obesity and associated conditions like diabetes and heart disease (Gao et al., 2023). Opting for more affordable options in the vicinity of campus, students choose to eat out rather than at the campus facilities. This behavior makes them prone to deficiency of nutrients is validated by the study highlighting insufficient intake of nutritious food ranks among the top six causes of health-risk behaviors reported among university students. ^5^

A report by WHO (2015a) states that ‘Healthy diets optimize growth and development over the short- and long-term. They are characterized by being sufficient and balanced in quantity and quality, containing a diversity of nutrient-dense foods including vegetables, fruits, whole grain cereals, fish, legumes, nuts, modest amounts of animal-source foods, limited in foods and drinks high in saturated and trans fats, added sugars and salt”. ^1^ A past study ^2^ has reported that WHO guidelines with respect to dietary habits are rarely practiced by students during their stay at the campus. A study conducted in Kansas in the USA found that a majority of the university students were obese and that their dietary habits needed to be changed to include more nutritional foods rich in fruits, vegetables, and fiber ^3^ Further, the study also recommended physical activity for the students. Consumption of a low proportion of fruits and vegetables and a high proportion of food having high calories, saturated fats, alcohols, and added sugar was reported among large number of university students. ^6^ Many studies in the past have pointed out that university students don’t follow nutritional-rich lifestyles. ^7^ Further, students find process of choosing food challenging, ^4^ hence, they gradually move away from healthy food habits. ^5^ Studies also show that only 4% of students consume 30% or less of energy from fat and 10% or less from sugar per day. ^3^ Hence, there is pressing need to inculcate healthy eating habits amongst the students especially in the face of the upsurge in lifestyle diseases. There is an urgent need to inspire nutrient-rich food behaviors amongst the students such that they select whole grains over processed food, include of fruits and vegetables in their daily diet and avoid health-compromising food choices. ^9^

A review study in the Indian context revealed that a substantial percentage of young people have unhealthy food habits that affect their overall progress. The issues of failure in achieving academic goals are on a rise, mostly interconnected to unhealthy food habits and are likely to intensify in the future. This study considers prime unhealthy habits as “under and over nutrition, common mental issues, non-communicable diseases (NCDs), and stress and anxiety commonly associated with current “nutrition and epidemiological transition”. ^10^

The present study attempts to apply Ajzen’s (1991) theory of planned behavior to comprehend and conceptualize the process by which students can shift their dietary preferences towards healthier options within the university campus. The theory of planned behavior (TPB) was proposed by Icek Ajzen in 1985 and is one of the most applied models to study the intentions and consumer behavior in various setting including food consumption. ^11^ The TPB states that behavior is influenced by intention to participate in that specific behavior (consuming healthy food) and perceived control behavior (PCB), for instance, practicing healthy food consumption at the campus irrespective of the environment. Intentions drive people to take conscious steps to perform a behavior. PCB is the ability of a person to control their behavior, in line with Bandurs’ concept of self-efficacy. ^12^ Terry et al., conducted a study among 146 undergraduate students using regression analysis and found that past behavior experiences (feedback) considerably foretell both, intentions and actual behavior. ^13^ In the present qualitative study, participants had to compulsorily subscribe to UDF during their first year of undergraduate program as per university policy. The postgraduate students were not restricted by policy. The approach to investigating previous behaviors (experiences) involved studying the period during which students engaged in the practice of selecting food choices starting from their first year, and examining how this process contributed to the development of feedback initiated repetitive behavior. This study stands out due to its utilization of a qualitative approach in examining the subject. In contrast, previous studies have solely employed a quantitative approach to investigate behaviors related to food consumption. ^14^

## Methods

A private university in Udupi was selected for this study since it has four UDFs on its campus. The university attracts students from all over the country with varied food habits. Second, first-year students dine at the UDF for one year. For the study, the duration of one year and the consecutive years thereafter were considered integral to developing feedback on UDF food. Accordingly, the undergraduate students of year II-IV and post-graduate students of the university were selected as part of this study. This research employed the qualitative research method and interpretive phenomenologic approach to analyze data and glean insights. The snowball sampling method was applied to recruit participants. An in-depth interview method was used to gather data and gather more subjective views. The interviewer did not have any contacts or relations with the participants before the interview. Students of the engineering college were contacted first and requested to participate in study. As per inclusion criteria, they had to be residents of the university accommodation, with their ages ranging from 18+ to 24 years and pursuing year II through year IV of their study programs. The first-year students pursuing undergraduate programs were excluded from the study due to their below 18 age. First, a few participants were selected by purposive sampling who then recommended other potential participants from their WhatsApp groups. Undergraduate students from years II through IV and post-graduate from years I and II of their study program were invited to participate in the study via their WhatsApp groups. Their participation was voluntary, their verbal consent was taken for participation, and the interview time was confirmed in advance. The participants were not involved in the study beyond data collection and transcripts were only discussed with the research team.

The ethics clearance certificate was obtained from Kasturba Medical College and Kasturba Hospital Institutional Ethics Committee before recruiting the participants for the study. An in-depth interview guide was prepared and validated by the doctoral committee for PhD study and experts from nutrition, culinary arts, and qualitative research fields. A pilot study was conducted to test the interview guide and based on the findings, the necessary modifications were incorporated. Participants’ consent was verbal as they were interviewed online via the MS Teams platform and the interaction was audio-recorded based on their consent. Online interviews were conducted with twenty-six students belonging to various regions of India and living on the campus. Data was collected until saturation. Two students couldn’t participate because they had to appear for their examinations. Only, the research team was present during the online interviews with students who were at their place of residence due to the pandemic lockdown.

The interviews were conducted in English and then transcribed. Using the deductive approach, the codes were identified and grouped under code families. The data were coded manually ^15^ and analyzed by thematic analysis using the Atlas.ti data management software. Coding was performed by the primary researcher and one other research team member. Following this, the codes were reviewed by the research team. Coded data were grouped according to relevance to enlist categories. This helped us to create themes and sub-themes. The themes derived were discussed with the research team members and consistency between data and findings was established.

## Results

**Figure 1.**
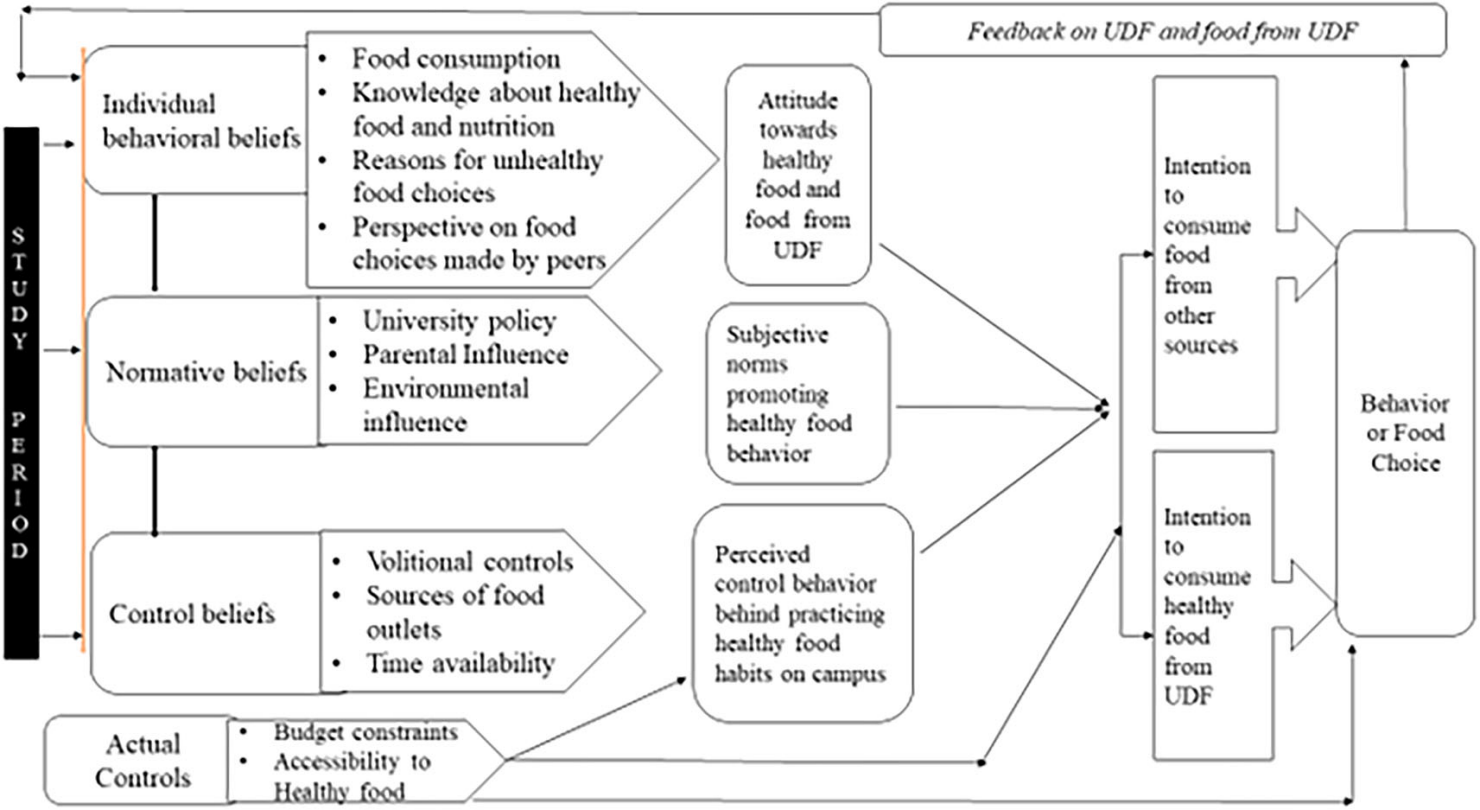
Conceptual model of food choice.

The demographic profile of the participants revealed that students were pursuing courses in engineering, architecture, medicine, dental, pharmacy, and forensic medicine. Their age ranged between 18 to 24 years and most belonged to Mumbai, Kolkata, Ahmadabad, Karkala, Ghaziabad, Bangalore and Kochi. Figure 1 shows the conceptual model of the food choices of the students that emerged after data analysis.

The conceptual model was developed by applying the theory of planned behavior. It included the following components 1. Individual behavior beliefs 2. Normative beliefs 3. Control beliefs 4. Actual controls, and 5. Feedback. The continuous interdependency and relationship of beliefs about UDF food by participants led to attitude development towards healthy food which, in turn, initiated the transition in intentions to opt for UDF food followed by actual behavior. This cyclic process was observed throughout their stay at campus including the effect of feedback on behavior (food choice) and, accordingly, transition in food choices. Each component of the conceptual model is described using examples from data.

## Individual behavioral beliefs

Individual behavior beliefs were developed based on participants’ experience with respect to their food consumption routines, knowledge about healthy food, views on nutrients requirements, perceptions about the after-effects of adopting unhealthy food choices and their observations of food choices of their peers. Individual beliefs motivated the development of attitude and intention to adopt healthy food choices.

### Food consumed on campus

The data analysis established that all the participants were aware of the need to consume healthy food and practiced healthy food consumption whenever possible. P10-“ *I eat almonds. I have a box of almonds in my room, so I eat that when I am hungry or when I am studying, like maybe 20 – 30 I eat*” …. Data sample also showed that students made a conscious effort to add fruit and vegetable juices, almonds, boiled eggs or omelets, idly, and dosa (rice-based breakfast items) to their diet. P11, “I *have tried to make sure I take juice, or I go to the market and buy an apple, grapes or pineapple. When I became really conscious about my health, then I used to have oats.*”

Data also showed that UDF also served non-vegetarian food items (fish and chicken) as a protein source. Further, it also served vegetable juices, dals (pulses) based curries, and rice-based items in its main meals. P13-“ *The only reason I tolerate the mess dal is that I need proteins. And in the morning we get upma, bread/butter, dosa, idli and all these things. So carbs are there. And fibers, it’s mostly we get fibers when we eat fruits. And if I get a glass of milk I get my vitamins there.*” Our data confirmed that the students were aware of the importance of consuming nutrient-rich food, and how important this was to keep healthy and achieve academic success. They also had a positive attitude towards UDF and the food it served because it included nuts, fruits, vegetables, and proteins.

### Healthy food and knowledge about nutrition

Data analysis revealed that participants were well aware of the fact that the UDF served “balanced and wholesome meals”. P7- *“I prefer fresh food, freshly cooked food”*

P8-“ *For daily nourishment, I prefer the mess (UDF) food. So I actually try to eat my meals in the mess (UDF), as much as possible, because they provide quite a wholesome diet*”.

Our interactions with the students revealed that they made a conscious attempt to consume fresh fruits and nuts along with meals, avoid food from restaurants, junk food/fast food, ready-to-eat food, and fried food. Besides, they also perceived rice to be healthier than noodles. However, they lacked knowledge about the nutritional value of the food items and this was a barrier in adopting healthy food practices.

### Food consumption behavior of peers

Food consumption habits of peers greatly affected individual food choices. Our data confirmed the belief that the peers made unhealthy food choices based more on taste than the actual nutritional value of the food. Peer influence had a great role to play in developing the food culture at the campus. P3-“ *In my friend circle, there are only 20 to 30% students who are really conscious, and about I would say 30 to 40% are at least a little conscious about healthy food.*” Around 60% of the population was indifferent to nutrient rich food and mostly consumed food based on taste, accessibility and convenience (quick service food). P4-“ *They go more for the taste, they want more spicy gravies or more paneer or more of pasta. Something tastier, and they don’t care if the food is nutritional or not. It has to be tasty, this is a primary objective.*” The campus population was categorized into different groups based on their food behavior. About 20-25% peers inconsistently practiced healthy food habits, followed a certain diet, exercised regularly but preferred cheat diets on weekends. About 10-12% peers practiced strict diets, regularly participated in physical activity followed fad diets, but were found to be consuming fewer nutrients than required. P 20-“ *My experience now people are becoming health conscious, some people are fitter and there are others who starve themselves, and take less nutrients. This generation doesn’t know, they don’t know how much the body needs, how much intake is required, how to take it, and they lack this information*”. Our data confirmed that there was an unhealthy food culture in the campus, mainly attributable to lack of proper knowledge and guidance about healthy food, and partly because many believed it to be part and parcel of campus life. Our data also showed that athletes or students involved in sports activities consistently practiced nutritious diet irrespective of their surroundings.

### Causes of unhealthy food choices

The participants expressed that during their first and second year of college they preferred to dine at the UDF because it served healthy and nutrient-rich food. P11-“ *There was this stage when I was into healthy food. But then, exams come and one month before exams, I had to start studying, so I stopped caring for my diet.”*

The participants expressed that their dietary habits took a turn for the worse as they progressed through they college life burdened with their increased academic responsibilities. Their lives became more disorderly leaving them little time to concentrate on their food habits and choices. P9- “ *I rarely get to sleep. My sleep schedule is … I sleep after 3 or 4 a.m. I eat some snacks like peanuts, or I eat noodles or a sandwich, or just have coffee*”. The lifestyle changes were induced by academic stress, time devoted to extracurricular activities, and long working hours, among other factors. P15-“ *Once I started eating dry fruits in the morning, however, with such busy schedules, on most days I just forgot to take them. But as we got more classes, we started missing more lunch breaks and it got more messed up. So that is why I had to start eating outside at odd hours.*”

P3-“ *I would drink milk daily. But my breakfast pattern has changed a lot since the last year, because I am so heavily involved in so many activities. I really don’t have the time to think about food and what I am eating.*” Our data, thus, confirmed that the food habits were greatly impacted by factors such as busy schedules, working at odd hours, skipping meals, high cost of healthy food, and accessibility to healthy food. On the other hand, cheap unhealthy food was easily available and so students gradually transitioned to adopting unhealthy food habits. P5-“ *Healthier options are more expensive and they are less tasty. So, I think students prefer food items that are both cheap and tasty.”* P3-“ *The majority of the food I eat is for relaxation. That’s why I don’t bother about nutrition.”*

The students also followed undisciplined sleeping and wakening patterns and as a result, they either missed having breakfast or ended up picking up RTE (Ready to Eat) food on the way to their classes or activities. P4-“ *Students usually skip breakfast because they wake up late. If they have to rush for class, they will pick up a roll, or a packet of biscuits or a packet of chips*”. Thus, factors influencing their food choices included academic stress, examination stress, lack of disciplined lifestyle, desire to experience new foods, convenience, financial constraints, easily accessibility of unhealthy foods, inclination to consume non-vegetarian food, and lack of accessibility to outlets offering healthy foods in comparison to the unhealthy options.

## Normative beliefs

Participants’ normative beliefs of subjective norms to promote healthy food behavior on campus included university policy, parental influence, and environmental influence. The subjective norms were seen to have positive impact students’ favorable attitude towards the UDF.

### University policy

Participants expressed that the university conducted awareness programs on adopting healthy food habits and culture. P2-“ *1 am gaining some knowledge about nutrient contents of various food items. 2. We gain knowledge about specific nutrients that we benefit from these foods, 3. We need to educate the people about the nutrients we should consume.”*

Students expressed that it would help if the university made some changes to the food offered at the UDF based on their nutrient quality and taste. This would help to develop a positive attitude towards UDF.

P15- “ *Then a little bit of motivation required from our side only, some motivation is required for us to eat fruits and take juices etc.*”

Participants also expressed that they were aware of lifestyle diseases like diabetes and obesity that are now prevalent amongst young people and mostly caused by unhealthy dietary habits. P9- *I think it’s the diseases, it’s the health problems that everyone is getting. I think it is quite visible in the young generation. People are getting diabetes, or obesity problems and many hormonal problems also. And I think it’s the eating habits. I think the only solution to this is to have at least the basic nutrition in our food.* Students also expressed that it would greatly help if the university regularly sent out notifications to motivate students to adopt healthy food habits. P20-“ *How much nutrients body needs, some people take too much some people take less. The authorities must inform us that this is a requirement and this is the way to get it. Actually nobody knows, nobody calculates, how much intake is required. May be making charts, making dining at the mess (UDF) mandatory would help. Outside food we should not consider, this food has this much nutrition, your body needs this, make posters, charts, PPTs to create awareness.”* Participants also believed enriching junk food with nutrients could help to kill two birds with one stone, that is, achieve nutrient value without compromising on taste. P17-“ *We have to increase nutrients in junk food (laugh), students will eat.*” Further, improving taste and quality of the UDF food would motivate transition to healthy food.

*Parental influence*

Participants expressed that parents had a great role to play in influencing their food consumption habits. P9-“At *home we can all get nutrition. Because when we are in the hostel, not everything is accessible. So while we were there, if we just have some biscuits that are sugary, like Milkbikis or something, at home we have an option to choose. My dad has turned me into a more nutritional-conscious person.*” Parental influence from childhood to adulthood has a great role to play in inculcating healthy food habits for life.

P6-“Because my mother has made sure I have almonds daily since I went to school so I have almonds in my hostel room. I eat regularly, Almonds or pistachio.”

P4 *-“I mean, there are more different kinds of vegetables at home. At home there are more kinds of sabzis (vegetables) and so we don’t consume chips and such stuff.”*

*Environmental influence*

Participants further expressed that the university could leverage social media to promote healthy food culture in the campus environment. P9-“ *Some socially active programs or something can help. Maybe they should be attractive enough to draw attention. Some innovative messaging is required.”*

They also felt that student clubs can play a more proactive role in this direction through education, awareness and listing facilities outside the campus where they could purchase healthy food at reasonable rates. P9 *-“I think, when I actually see that there is a place where we can access food, I think I would try it. I think young people would.*”

Participants realized the importance of physical activity along with consuming healthy food.

## Control beliefs

The preconceived notions about food greatly influenced the students’ transitional journey towards healthy food irrespective of peer pressure and their social circle during their stay at the campus.

### Volitional controls

It was observed that some of the respondents were not distracted by their immediate environment and maintained steady food habits. P18-“ *Everyone is going for junk food, but I think there are still some people who are health conscious.*” Several respondents were alive to the importance of adopting healthy food habits to improve their academic performance. P15-“ *We have full-days of classes and we need to maintain our energy levels. And when you tend to eat healthily, you fall less sick. So it is also very important to maintain good health.”*

Commenting on why some students were indifferent to eating healthy, P3 said, “ *It depends on their interests. If they think that their fitness is a priority, then automatically they will be conscious.*”

P5-“ *I participate in events and competitions, I do ultra-running, like 60 km. So, I trained as an athlete, long distance. I never skipped vegetables.”*

According to the participants, sports people and athletes were more prone to make healthy eating choices because for them winning was at stake. Their determination to win was a motivation to exhibit disciplined food consumption behavior.

### Sources of food outlets on campus

Students were aware that their university campus offered several food outlets comprising of university dining facilities and other food joints or canteens selling both freshly cooked food and ready to-eat food (RTE), along with confectionary. P15-“ *When you are under stress you tend to binge eat. You buy whatever you get, the available packet food or whatever you are craving for.*” Participants also admitted that they were dependent on the other food joints when they were unable to dine at UDF. Usually, for late-night snacks participants frequently visited retail outlets as they were closer to their hostels.

### Time availability

Most participants expressed that they were pressed for time due to their over busy schedules. As a result, they consumed whatever they could easily lay their hands on rather than go out of the way to hunt for high nutrient food. P19-“ *College timings, how far is college, if we have immediate class after lunch, then we cannot go out, these factors affect what we eat, and so we end up eating some egg roll, that should happen only once a while actually*.”

Post-data analysis showed that students were hard-pressed for time because they were far too focused on their studies, preparing for exams, and late night study to better their performance. Further, all these factors together affected their sleeping and waking habits. As a result, they had little time to prioritize their health and healthy eating habits.

### Actual controls

Actual controls include means to accomplish behavior. Our data analysis showed that students were faced with budget constraints and lack of easy accessibility to healthy food outlets. Actual controls influenced their intentions to practice healthy food habits.

### Budget constraints

The students also expressed that budget was a constraint and so they didn’t have adequate funds to purchase high quality, nutrient-rich food. So they preferred to dine at the UDF during the last week of the month and explore other food options around the campus for rest of the month. P12-“ *When out of money, I eat over there (UDF).”*

P5-“ *Healthier options are more expensive or less tasty. So, I think students focus on taste rather than nutritive value.*”

Our data analysis showed that students believed that nutritious food was heavy on their pockets and so they preferred to buy the unhealthy options available. Second, taste was another deciding factor motivating the purchase of non-healthy food.

### Accessibility to healthy food

Participants also expressed that unhealthy options were more readily available near he campus than the healthy ones. P9-“ *If there is shop having healthy snacks nearby the place of residence, I think I would try it.*” *P15-UDF provides a proper balanced diet, but people opt out of the mess (UDF) because the food is not that tasty.”* For them, UDF was the only facility offering healthy food within their budget. They opined that if the UDF improved the taste and quality of the food it served, they would gladly transition to healthy eating.

## Feedback on UDF (post-behavior)

The Theory of Planned Behavior (Ajzen, 1991) describes “Feedback” as the post-behavior understanding of the actions taken (Behavior). Studying the current problem from the lens of the theory of planned behavior, it was found that the students displayed their intention choose UDF over other sources to access healthy food based on feedback.

### Nutritional quality and Credibility of UDF

Examination of data testified that the UDF served nutrition-rich food, a fact that was confirmed by the respondents. P21-“ *Because they have divided nutrition of everything, they are performing their duties well.”* While the UDF offered a balanced diet, the respondents expressed their concerns about the small portions of protein-rich food items that were served and the fact that the facility did not disclose the nutritive value of the foods that could add to their awareness. P24-“ *Whatever they provide in mess (UDF) is nutritive. They provide dals and lentils which are nutritive. It’s completely fine, the food is nutritive*.”

A few participants doubted the credibility of the UDF perceiving it as more business-oriented and comprising quality for cost. P7- “ *What they are mainly trying to do is to cut costs wherever they can. So it is not exactly for the students benefit.*”

The students expressed their dissatisfaction with the UDF for not disclosing the nutritive values of the food it served the preparation methods it followed, and the absence of a nutritionist who could provide the necessary advice. As a result, these students preferred to dine out than in the facility. However, on the whole, a majority of the respondents were satisfied by the food and services provided by the UDF.

*Sensory beliefs of food from UDF*

Participants expressed their dissatisfaction with the sensory experience the UDF food provided, expressing that it lacked taste. P15-“ *The mess people provide properly balanced diet, but people opt out of the mess because of the taste. So if the taste factor is improved, people might stay with the mess and get some nutritious food.”*

Most participants agreed that UDF food needs improvement. They found the curries to be too oily, the food lacked spices, the quality of chapattis was poor, and there were too many potatoes and less greens in the menu.

## Discussion

The conceptual model developed for this study is in line with the theory of planned behavior (Ajzen, 1991), and grounded in the students’ perspectives about the UDF food and their own food choices. The model represents a comprehensive process of transition in food choices as they progressed through their stay at the campus. On average, the students stay on campus for two to four years and during this time their food habits change. Their behavioral beliefs, subjective norms and preconceived control beliefs impact their attitude towards UDF and their perceptions about the quality of the food it serves. In the first year, the students are required to dine at the UDF, post which they begin to explore outside options driven by several factors. This is substantiated by the respondents who expressed that they dined at the UDF during the first two years of their study after which many of them explored other options. Most respondents expressed their dissatisfaction with the taste, and this was one of the primary causes for their exploring other avenues even though they served unhealthy food. ^21^ While the food lacked in nutrition it served as a source of relaxation. This finding was in line with another study. ^22^ It was also found that taste was a major factor driving transitions in food habits. Further, the study also found that taste as a sensory experience varied from one person to another. ^22^ Thus, taste is unique to each person and so it is difficult to identify which food has higher sensory attributes. Hence, sensory appreciation cannot be a means to predict acceptance of a certain food item. ^23^

In line with past studies, the current study also found that the factors that impacted food choices were taste, ^27^^,^^28^ price, ^16^ time availability, ^17^^,^^18^ convenience (Maillet & Grouzet, 2023), academic stress, ^18^ lack of knowledge about nutrition, ^19^^,^^30^ (Gao et al., 2023), absence of display boards notifying nutrient content of each meal, ^31^ and cheap cost of unhealthy food ^17^ compared to healthy foods. ^6^ However, the study adds new factors to the literature, namely, the concern expressed by the respondents in terms of lack of a nutritionist at the UDF to guide and advice about the food served at the UDF, and their lack of knowledge on how the food was prepared at the facility. The students also expressed their concerns on the lack of other outlets selling healthy food on the campus. The reasons for deviation from healthy food choices were uniform irrespective of their year of study, education stream, and gender. ^6^ Further, the study also finds that the UDF needs to evolve its services, guided by the administration, and develop a healthy food culture on the campus that is appreciated by the students. ^20^ This recommendation is in line with past research, which emphasized the role of UDF as, “responding to young adult’s food preferences, food service cost-effectiveness, efficiency, and student’s health considerations”. ^32^

The participants expressed their concerns about the UDF which prompted them to explore other eating options. The theory of planned behavior (Ajzen 1991) states that feedback is necessary to bring about the necessary behavioral changes based on current behavior. ^11^ The conceptual model aptly illustrates the transition in food choices. According to this theory, “background factors,” such as age, education, religion, income, general attitude and emotions etc. indirectly influence a person’s intentions and behavior by effecting behavioral, normative and control beliefs. The data analysis draws insights on study program duration as one of the background factors which stimulated participants’ intentions and behavior towards food choices. The study finds that the respondents were positively disposed toward changing their attitudes and adopting healthy food habits. This is in line with other studies that have found that intentions are a precursor for accomplishing a particular behavior. Success in accomplishing behavior will not be impacted by the will or intention to perform the behavior but also by other control factors, such as requisite chances and means. These requisite chances, means and intention to perform behavior influence a successful transition towards the desired behavior. ^27^ This is well-illustrated in the conceptual model in which these controls are in the form of volitional controls and budgetary constraints affecting students’ intentions and behavior.

The study establishes a relationship between a healthy environment and healthy food choices ^24^ in which the environment facilitates the accessibility and consumption of fruits, vegetables and nutrient-rich food. ^25^ Students could be made aware of the need to consume healthy foods and display the nutritive value chart of the foods served in the UDF in the facility itself. Further, affirmative messages can be shared regularly in the form of posters, charts, digital boards flashing PPTs through social media and other messaging platforms, and other outreach activities. ^26^ Similar findings were noted in other studies also. ^33^

The model was conceptualized developed based on the in-depth understanding achieved through the study and not the scale of sample. To achieve this, specific university students from diverse regions and studying at same university campus were invited to share their food experiences. The sample did not aim to represent the students’ population; rather, it was aimed to identify the factors that motivated them to change their food choices over the period of their stay at the campus. Our study sample included students from varied social backgrounds and exposed to different food cultures, with the aim to understand their food transition journeys. The model components display food events held throughout the duration of their stay and the role of feedback in developing an attitude. Future works can consider participants belonging to different universities, geographical locations, and educational streams. An insight on significance of UDF and its role in a students’ transitional food journey was not exclusively objective-oriented but emerged through food events or actions experienced by participants. However, the role of UDF may vary depending on the geographical location of the university.

## Conclusion

The conceptual model developed to understand food choices focuses on food actions exhibited by individual participants and their overall impact on formation of an individual’s attitude towards healthy food consumption. The created model provides decision-makers in universities with valuable information on encouraging students to choose healthy foods, thereby improving their academic performance. Moreover, the UDF must adapt its services to align with the nutritional needs of the students while ensuring that taste is not compromised or additional costs are not incurred. Past studies have shown that a conducive environment promotes healthy food habits. The universities can ensure an ideal environment by ensuring easy availability and accessibility to healthy foods. ^6^^,^^25^ The conceptualized model projected in the paper is a first of its kind attempt to understand what governs students’ food choices. Further, research can consider diverse participants in diverse settings.

### Limitations

Food habits studies are multidimensional, individualistic and circumstances-oriented ^4^ for which specific theory or perspective may not be possible. The participants in the present study were students from technical and health science programs representing a small cross-section of the actual student population in the university. As students’ progress to final years of graduation program they tend to live in off-campus apartments. ^34^ These students too were not considered in the study and can be subject to future research. Another limitation of the study was that the interviews were carried out online, and we may have missed the opportunities provided by face-to-face interactions.

## Data availability

Figshare. DataPerceptions.docx. DOI: https://doi.org/10.6084/m9.figshare.20766088.v1 ^35^

This project contains the following underlying data:

- Data is transcribed interviews.

Data are available under the terms of the Creative Commons Attribution 4.0 International license (CC-BY 4.0).
