# Conceptualizing university students’ food choices based on theory of planned behavior

**DOI:** 10.12688/f1000research.123325.3

**Published:** 2025-03-07

**Authors:** Kshama Vishwakarma, Varalakshmi Chandra Sekaran, Vidya Patwardhan, Asha Kamath

**Affiliations:** 1 Associate Professor, Masters in Personal Management, DHMCT, Welcomgroup Graduate School of Hotel Administration, Manipal Academy of Higher Education, Manipal, Karnataka, 576104, India; 2 Associate Professor, Global Public Health Policy and Governance, Prasanna School of Public Health, Manipal Academy of Higher Education, Manipal, Karnataka, 576104, India; 3 Professor & Coordinator, Centre for Hospitality and Tourism Research, Welcomgroup Graduate School of Hotel Administration, Manipal Academy of Higher Education, Manipal, Karnataka, 576104, India; 4 Department of Applied Statistics and Data Science, Prasanna School of Public Health, P Manipal Academy of Higher Education, Manipal, Karnataka, 576104, India

**Keywords:** Food choices, university students, campus, university dining facility (UDF), qualitative research, healthy food, theory of planned behavior, food transition.

## Abstract

**Background:**

Higher education at the university level is essential for advanced learning, enhancing academic knowledge, and precipitating significant life changes. These include lifestyle adjustments, relocation from one’s primary residence, and the acquisition of autonomy in decision-making. Numerous students opt to reside in dormitories, resulting in notable alterations in dietary habits. Campus cuisine differs substantially from their previous domestic diets, potentially influencing their overall growth and development during their academic tenure.

**Method:**

The study uses the theory of planned behavior to conceptualize and understand university students’ food choices, employing qualitative research and a phenomenological approach. Snowball sampling selected 26 undergraduate and postgraduate students in technical and health science programs from a private university in Udupi who were residing on campus for two to four years. Online interviews were audio-recorded with participants’ consent.

**Results:**

Transcribed interviews were coded and categorized to identify themes, which were subsequently conceptualized to develop a model based on the theory of planned behavior. The duration of students’ campus residence provided insight into their perspectives on food events and consumption at the university dining facility (UDF), influenced by factors such as palatal preferences, cost considerations, temporal constraints (during academic activities), accessibility of nutritious options, academic-related stress, and insufficient nutritional knowledge. Two primary findings emerged: first, there was an absence of nutritional information displayed in the dining facility; second, students exhibited a preference for consuming less healthy food options off-campus due to their lower monetary cost.

**Conclusion:**

The investigation provides insights into the role of UDF in offering nutritionally balanced meals to students, potentially contributing to improved health outcomes and enhanced academic performance. This research elucidates the relationship between students’ dietary choices and their subsequent impact on scholastic achievement.

## Introduction

Students pursuing higher education frequently utilize residential facilities provided by their colleges and universities. While residing in a novel environment, students experience significant alterations in their lifestyle, deviating from the established habits maintained in their home environments. ^1^ The transition to university frequently encompasses the formation of new social connections, exposure to novel experiences, and personal growth. Significantly, this transition coincides with a critical period for establishing enduring habits, ^2^ including a crucial change in dietary preferences. ^1^ An individual’s dietary practices and beliefs during their tertiary education years can significantly influence their habits in adulthood, potentially affecting the probability of developing obesity and associated conditions, such as diabetes and cardiovascular diseases. ^3^ Students opting for more economical dining options in proximity to campus choose to consume meals outside the campus facilities. This behavior, which predisposes them to potential nutrient deficiencies, is corroborated by a study highlighting that insufficient intake of nutritious food ranks among the top six health risk behaviors reported among university students. ^4^

A report by the WHO (2015a) ^5^ states that “healthy diets optimize growth and development over the short and long term. They are characterized by being sufficient and balanced in quantity and quality, containing a diversity of nutrient-dense foods including vegetables, fruits, whole grain cereals, fish, legumes, nuts, modest amounts of animal-source foods, limited in foods and drinks high in saturated and trans fats, added sugars, and salt”. ^5^ A past study ^6^ has reported that students rarely practice WHO guidelines with respect to dietary habits during their stay on campus. A study conducted in Kansas in the United States of America revealed that a significant proportion of university students were classified as obese, and their dietary patterns necessitated modification to incorporate more nutritionally dense foods, particularly those rich in fruits, vegetables, and dietary fiber. ^7^ Furthermore, a low proportion of fruit and vegetable consumption and a high proportion of food containing elevated levels of calories, saturated fats, alcohol, and added sugar were reported among a significant number of university students. ^8^ Many past studies have pointed out that university students do not follow nutritionally rich lifestyles. ^9^ Further, students find choosing food challenging; ^1^ hence, they gradually move away from healthy food habits. ^4^ Studies also show that only 4% of students consume 30% or less of energy from fat and 10% or less from sugar daily. ^7^ Therefore, there is a pressing need to cultivate healthy eating habits among students, particularly considering the increasing prevalence of lifestyle diseases. It is imperative to promote nutrient-rich food behaviors among students, encourage them to select whole grains over processed food, incorporate fruits and vegetables into their daily diet, and avoid health-compromising food choices. ^10^

A review study in the Indian context revealed that a substantial percentage of young people have unhealthy food habits, which affect their overall progress. The issues of failure in achieving academic goals are on a rise, mostly interconnected to unhealthy food habits, and are likely to intensify in the future. This study considers prime unhealthy habits as “under and over-nutrition, common mental issues, non-communicable diseases (NCDs), and stress and anxiety commonly associated with current “nutrition and epidemiological transition.” ^11^

The present study aims to apply Ajzen’s (1991) theory of planned behavior to understand and conceptualize how students can modify their dietary preferences towards healthier options within university campuses. The theory of planned behavior (TPB) proposed by Icek Ajzen in 1985 is one of the most widely used models for examining intentions and consumer behavior in various contexts, including food consumption. ^12^ The TPB states that behavior is influenced by intention to participate in that specific behavior (consuming healthy food) and perceived control behavior (PCB), for instance, practicing healthy food consumption at the campus irrespective of the environment. Intentions drive people to take conscious steps to perform a behavior. PCB is the ability of a person to control their behavior, which is in line with Bandurs’ concept of self-efficacy. ^13^ Terry et al. conducted a study of 146 undergraduate students utilizing regression analysis and found that past behavioral experiences (feedback) significantly predicted both intentions and actual behavior. ^14^ In the present qualitative study, participants had to compulsorily subscribe to UDF during their first year of undergraduate program as per university policy. Postgraduate students were not restricted by policy. The rationale for selecting qualitative methodology was that studies have demonstrated that specific societal groups exhibit diverse attitudes, motivations, and behaviors concerning healthier eating habits. ^15^ In-depth interviews ^16^ provide a methodological tool for investigating food choices from an individual perspective and allow for in-depth study of the problem. The approach to investigating previous behaviors (experiences) involved examining the period during which students engaged in selecting food, commencing from their first year, and analyzing how this process contributed to the development of feedback-initiated repetitive behavior. This study uses a qualitative approach to examine this subject. In contrast, previous studies exclusively employed a quantitative approach to investigate behaviors related to food consumption. ^17^

## Methods

A private university in Udupi was selected for this study because it has four UDFs. Universities attract students from all over the country with varied food habits. Second, first-year students dine at the UDF for one year. For this study, the duration of one year and consecutive years thereafter were considered integral to developing feedback on UDF food. Accordingly, undergraduate students in years II to IV and postgraduate students from the university were selected. As Creswell (2018) ^18^ asserted, phenomenological studies describe the common meaning of a phenomenon being studied by all participants. “It seeks to reduce individual experiences to a description of a universal essence”. ^18^ Hence, this study employed a qualitative research method and interpretive phenomenological approach to analyze the data and gain insights. Snowball sampling was used to recruit the participants. An in-depth interview was used to gather data and provide more subjective views. The researcher had no prior contact or relationship with the participants before conducting the interviews. Students from the engineering college were initially contacted and invited to participate in this study. According to the inclusion criteria, they had to be residents of the university accommodation, with ages ranging from 18+ to 24 years, and pursuing year II through year IV of their study programs. First-year undergraduate students were excluded from the study because they were younger than 18 years old. Initially, a subset of participants was selected through purposive sampling, who subsequently recommended additional potential participants from WhatsApp groups. Undergraduate students from years II through IV and postgraduate students from years I and II of their respective study programs were invited to participate via WhatsApp. Participation was voluntary and verbal consent was obtained before participation. The interviews were conducted in advance. Participants’ involvement was limited to data collection and transcripts were discussed exclusively within the research team.

An ethics clearance certificate was obtained from the Kasturba Medical College and Kasturba Hospital Institutional Ethics Committee before recruiting participants for the study. The doctoral committee approved an in-depth interview guide for PhD studies, and experts from nutrition, culinary arts, and qualitative research fields validated the same. A pilot study was conducted to evaluate the interview guide, and necessary modifications were implemented based on these findings. Participants provided verbal consent as they were interviewed online via the MS Teams platform, and the interaction was audio-recorded with their consent. Online interviews were conducted with twenty-six students from various regions of India residing on campus. Data collection continued until saturation was achieved. Two students were unable to participate because of their commitment to the examination. The research team was present during online interviews with students who were at their places of residence because of the lockdown. The interviews were conducted in English and transcribed.

Data processing was performed using the deduction approach. Codes were identified and categorized into code families. The data were manually coded ^19^ and analyzed through thematic analysis using atlas —ti data management software. The primary researcher and an additional research team member performed coding. Subsequently, the research team reviewed these codes. The coded data were grouped according to their relevance to establish categories. This process facilitated the development of themes and sub-themes. The derived themes were discussed with the research team members and consistency between the data and findings was established.

## Results

**Figure 1.**
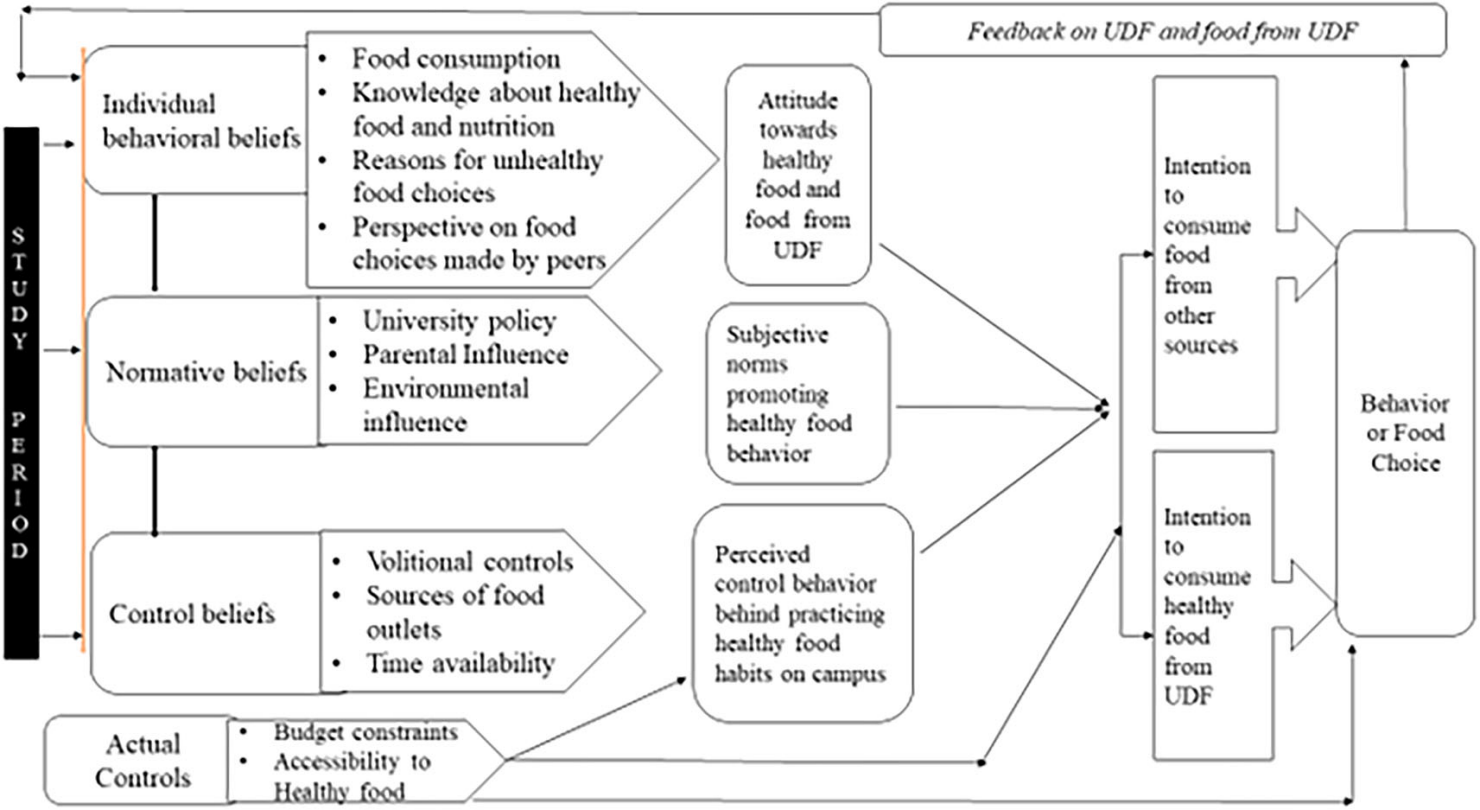
Conceptual model of food choice.

The demographic profile of the participants revealed that they were pursuing engineering, architecture, medicine, dental, pharmacy, and forensic medicine courses. Their ages ranged from 18 to 24 years, and most of them belonged to Mumbai, Kolkata, Ahmadabad, Karkala, Ghaziabad, Bangalore, and Kochi. Figure 1 shows the conceptual model of the students’ food choices that emerged after the data analysis. Systematic data analysis was used to identify the factors influencing participants’ food preferences. The themes developed through this analysis were organized to align with the principles of the theory of planned behavior and conceptualized according to the theoretical model.

The developed conceptual model included the following components: 1. Individual behavior beliefs 2. Normative beliefs 3. Control beliefs 4. actual controls, and 5. Feedback. The continuous interdependency and relationship of beliefs regarding UDF food among participants led to the development of attitudes towards healthy food, which, in turn, initiated a transition in intentions to select UDF food, followed by actual behavioral changes. This cyclical process was observed throughout their campus residence, including the effect of feedback on behavior (food choice), and consequently, transitioning into food selection. Each component of the conceptual model was elucidated by using examples from the data.

### Individual behavioral beliefs

Individual behavioral beliefs were developed based on participants’ experiences with respect to their food consumption routines, knowledge about healthy food, views on nutrient requirements, perceptions about the after-effects of adopting unhealthy food choices, and their observations of the food choices of their peers. Individual beliefs motivated the development of attitudes and intentions toward adopting healthy food choices.

### Food consumed on campus

The data analysis established that all participants knew the need to consume healthy food and practiced it whenever possible. P10-“ *I eat almonds. I have a box of almonds in my room, so I eat that when I am hungry or studying, like maybe 20 – 30 I eat.*” The data sample also showed that students consciously added fruit and vegetable juices, almonds, boiled eggs or omelets, idly, and dosa (rice-based breakfast items) to their diets. P11, “I *have tried to make sure I take juice or go to the market and buy an apple, grapes or pineapple. When I became conscious about my health, I used to have oats.*”

The data also showed that the UDF served non-vegetarian food items (fish and chicken) as a protein source. Furthermore, it also served vegetable juices, dals (pulses)-based curries, and rice-based items in their main meals. P13-“I only tolerate the mess dal because *I need proteins. In the morning, we get upma, bread/butter, dosa, idli, and all of these things. So carbs are there. We mostly obtain fibers when we eat fruit. And if I get a glass of milk, I get my vitamins there.*” Our data confirmed that students knew the importance of consuming nutrient-rich food and its importance in maintaining health and achieving academic success. They also had a positive attitude towards UDF and the food they served because it included nuts, fruits, vegetables, and proteins.

### Healthy food and knowledge about nutrition

The participants demonstrated limited knowledge regarding the daily nutritional requirements obtainable through an appropriate diet, and believed that freshly prepared food represents the optimal approach to managing their nutrient intake.

Data analysis revealed that participants knew the UDF served “balanced and wholesome meals.” P7- *“I prefer fresh food, freshly cooked food”*.

P8-“For *daily nourishment, I prefer the mess (UDF) food. So, I try to eat my meals in the mess (UDF), as much as possible, because they provide quite a wholesome diet*”.

Our investigation of student behavior revealed that participants deliberately incorporated fresh fruits and nuts into their meals while avoiding food from restaurants, processed food, ready-to-eat food, and fried food. They also expressed the perception that rice is a healthier alternative to noodles. However, the students demonstrated a lack of knowledge regarding the nutritious composition of various food items, which presented a significant barrier to adopting healthier dietary practices.

### Food consumption behavior of peers

Peer food consumption habits greatly affected individual food choices. Our data confirmed the belief that peers made unhealthy food choices based more on taste than on the actual nutritional value of the food. Peer influence plays a significant role in developing food culture on campus. P3-“In *my friend circle, only 20 to 30% of students are conscious, and about I would say 30 to 40% are at least a little conscious about healthy food.*” Approximately 60% of the population was indifferent to nutrient-rich food and mostly consumed food based on taste, accessibility, and convenience (quick-service food). P4-“ *They go more for taste; they want more spicy gravies, paneers, or pasta. Something is tastier, and they don’t care if the food is nutritional. It has to be tasty; this is a primary objective.*” The campus population was categorized into different groups based on their food behavior. About 20-25% of their peers inconsistently practiced healthy food habits, followed a certain diet, and exercised regularly but preferred cheat diets on weekends. About 10-12% of peers practiced strict diets, regularly participated in physical activity, and followed fad diets, but were found to consume fewer nutrients than required. P 20-“ *My experience is that people are now becoming health conscious; some are fitter, and others starve themselves and take fewer nutrients. This generation does not know; they do not know how much the body needs, how much intake is required, how to take it, and they lack this information*”. Our data confirmed that there was an unhealthy food culture on campus, mainly attributable to a lack of proper knowledge and guidance about healthy food, and partly because many believed it to be part and parcel of campus life. Our data also showed that athletes or students involved in sports activities consistently practiced a nutritious diet, irrespective of their surroundings.

### Causes of unhealthy food choices

Participants expressed that they preferred to dine at the UDF during their first and second years of college because they served healthy and nutrient-rich food. P11-“There *was this stage when I was into healthy food. But then, exams come, and one month before exams, I had to start studying, so I stopped caring for my diet.”*

The participants expressed that their dietary habits worsened as they progressed through college, burdened by increased academic responsibilities. Their lives became more disorderly, leaving little time to concentrate on food habits and choices. P9- “ *I rarely sleep. My sleep schedule is … I sleep after 3 or 4 a.m. I eat snacks like peanuts, or I eat noodles or a sandwich, or just have coffee*”. Lifestyle changes are induced by academic stress, time devoted to extracurricular activities, and long working hours, among other factors. P15-“ *Once I started eating dry fruits in the morning; however, with such busy schedules, I just forgot to take them on most days. However, as we got more classes, we started missing more lunch breaks, which got messed. So that is why I had to start eating outside at odd hours.*”

P3-“I *would drink milk daily. But my breakfast pattern has changed a lot since last year because I am so heavily involved in many activities. I don’t have the time to think about food and what I eat.*” Thus, our data confirmed that food habits were greatly impacted by factors such as busy schedules, working odd hours, skipping meals, high cost of healthy food, and accessibility to healthy food. On the other hand, cheap unhealthy food was readily available, so students gradually transitioned to adopt unhealthy food habits. P5-“Healthier *options are more expensive and less tasty. So, I think students prefer food items that are both cheap and tasty.”* P3-“The *majority of the food I eat is for relaxation. That’s why I don’t bother about nutrition.”*

Students also followed undisciplined sleeping and waking patterns. As a result, they either missed breakfast or picked up (RTE) food on their way to their classes or activities. P4-“Students *usually skip breakfast because they wake up late. If they have to rush for class, they will pick up a roll or a packet of biscuits or chips*”. Thus, factors influencing their food choices included academic stress, examination stress, lack of a disciplined lifestyle, desire to experience new foods, convenience, financial constraints, easy accessibility of unhealthy foods, inclination to consume non-vegetarian food, and lack of accessibility to outlets offering healthy foods in comparison to unhealthy options.

## Normative beliefs

Participants’ normative beliefs regarding subjective norms that promote healthy food behavior on campus encompassed university policies, parental influence, and environmental factors. These subjective norms were observed to exert a positive influence on students’ attitudes towards the UDF.

### University policy

The participants expressed that the university conducted awareness programs on adopting healthy food habits and culture. P2-“ *1 am gaining some knowledge about nutrient contents of various food items. 2. We gained knowledge about the specific nutrients that we benefit from these foods. 3. We need to educate the people about the nutrients we should consume.”*

Students expressed that it would help if the university changed the food offered at the UDF, based on their nutrient quality and taste. This would help develop a positive attitude towards UDF.

P15- “ *Then a little bit of motivation is required from our side only; some motivation is required for us to eat fruits and take juices, etc.*”

Participants also expressed that they were aware of lifestyle diseases such as diabetes and obesity, which are now prevalent among young people and are mostly caused by unhealthy dietary habits. P9: *I think it’s the diseases, it’s the health problems that everyone is getting. I think it is quite visible in the young generation. People are getting diabetes, or obesity problems and many hormonal problems also. And I think it’s the eating habits. I think the only solution to this is to have at least the basic nutrition in our food.* Students also expressed that it would greatly help if the university regularly sent out notifications to motivate students to adopt healthy food habits. P20-“How *much nutrients does the body need? Some people take too much; some people take less. The authorities must inform us that this is a requirement and this is the way to get it. Nobody knows, and nobody calculates how much intake is required. Maybe making charts and mandatory dining at the mess (UDF) would help. Outside food, we should not consider that this food has this much nutrition; your body needs this. Make posters, charts, and PPTs to create awareness.”* Participants also believed that enriching junk food with nutrients could help kill two birds with one stone, thereby achieving nutrient value without compromising taste. P17-“We *have to increase nutrients in junk food (laugh); students will eat.*” Furthermore, improving the taste and quality of UDF food would motivate the transition to healthy food.

***Parental influence***

The participants expressed that parents had a significant influence on their food consumption habits. P9- “At *home, we can obtain nutrition. This is because, when we are in the hostel, not everything is accessible. So, while we were there, if we just had some sugary biscuits, like Milkbikis or something, at home, we had an option to choose from. My dad has turned me into a more nutritional-conscious person.*” Parental influence from childhood to adulthood plays a significant role in inculcating healthy food habits.

P6- “Because my mother has made sure I have almonds daily since I went to school, I have almonds in my hostel room. I regularly eat Almonds or pistachios.”

P4 *- “I mean, there are more different kinds of vegetables at home. There are more kinds of sabzis (vegetables) at home, so we don’t consume chips and such stuff.”*

***Environmental influence***

Participants further expressed that the university could leverage social media to promote healthy food culture in the campus environment. P9- “Some *socially active programs or something can help. Thus, they should be sufficiently attractive to attract attention. Some innovative messaging is required.”*

They also felt that student clubs could play a more proactive role in this direction through education, awareness, and listing facilities outside the campus, where students could purchase healthy food at reasonable rates. P9 *-“I think when I see a place where we can access food, I think I would try it. I think young people would.*”

Participants realized the importance of physical activity and consumption of healthy food.

## Control beliefs

The preconceived notions about food influenced the students’ transitional journey towards healthy food, irrespective of peer pressure and social circle during their stay on campus.

### Volitional controls

Some of the respondents were not distracted by their immediate environment and maintained steady food habits. P18-“Everyone *is going for junk food, but I think there are still some people who are health conscious.*” Several respondents understood the importance of healthy eating habits in improving academic performance. P15-“We *have full days of classes and need to maintain our energy levels. When one tends to eat healthily, one falls less sick. So, it is also especially important to maintain good health.”*

Commenting on why some students were indifferent to eating healthy, P3 said, “ *It depended on their interests. If they think their fitness is a priority, they will automatically be conscious.*”

P5-“I *participate in events and competitions; I do ultra-running, like 60 km. So, I trained as an athlete, long distance. I never skipped vegetables.”*

According to the participants, sports people and athletes were more prone to making healthy eating choices because, for them, winning was at stake. Their determination to win was a motivation to exhibit disciplined food consumption behavior.

### Sources of food outlets on campus

Students were aware that their university campus offered several food outlets comprising university dining facilities and other food joints or canteens, selling both freshly cooked food and ready-to-eat food (RTE), along with confectionery. P15-“ *When you are under stress, you tend to binge on eating. You buy whatever you get, the available packet food or whatever you crave.*” Participants also admitted that they depended on other food joints when they could not dine in the UDF. Usually, participants frequently visited retail outlets for late-night snacks, as they were closer to their hostels.

### Time availability

Most participants expressed that they were pressed for time because of their busy schedule. As a result, they consumed whatever they could easily lay their hands on rather than going out of the way to hunt for high-nutrient food. P19-“College *timings, how far is college, if we have immediate class after lunch, then we cannot go out, these factors affect what we eat, and so we end up eating some egg roll, that should happen only once a while.*”

Post-data analysis showed that students were hard-pressed for time because they were far too focused on their studies, preparing for exams, and late-night studies to improve their performance. Furthermore, all these factors together affect sleeping and waking habits. As a result, they had little time to prioritize their health and healthy eating habits.

### Actual controls

Actual controls include means of accomplishing behavior. Our data analysis showed that the students faced budget constraints and a lack of easy access to healthy food outlets. Actual controls influenced their intention to practice healthy food habits.

### Budget constraints

The students also expressed that budget was a constraint, so they did not have adequate funds to purchase high-quality, nutrient-rich food. Therefore, they preferred to dine at the UDF during the last week of the month and to explore other food options around the campus for the rest of the month. P12- “When *out of money, I eat over there (UDF).”*

P5-“Healthier *options are more expensive or less tasty. So, I think students focus on taste rather than nutritive value.*”

Our data analysis showed that students believed that nutritious food was heavy in their pockets, so they preferred to buy unhealthy options. Second, taste was another deciding factor motivating the purchase of unhealthy food.

### Accessibility to healthy food

Participants also expressed that unhealthy options were more readily available near the campus than healthy options. P9-“ *If there is shop having healthy snacks nearby the place of residence, I think I would try it.*” *P15-UDF provides a proper balanced diet, but people opt out of the mess (UDF) because the food is not that tasty.”* For them, the UDF was the only facility offering healthy food within their budget. They opined that if the UDF improved the taste and quality of the food it served, they would transition to healthy eating.

### Feedback on UDF (post-behavior)

The Theory of Planned Behavior (Ajzen, 1991) ^12^ describes “Feedback” as the post-behavior understanding of the actions taken (Behavior). Studying the current problem from the perspective of the theory of planned behavior, it was found that the students displayed their intention to choose UDF over other sources to access healthy food based on feedback.

### Nutritional Quality and Credibility of UDF

Examination of the data confirmed that the UDF served nutrition-rich food, a fact confirmed by the respondents. P21- “Because *they have divided nutrition of everything, they are performing their duties well.”* While the UDF offered a balanced diet, the respondents expressed concerns about the small portions of protein-rich food items served and that the facility did not disclose the nutritive value of the foods that could add to their awareness. P24-“Whatever *they provide in mess (UDF) is nutritive. They provide dals and lentils, which are nutritive. It’s completely fine; the food is nutritive.*”

A few participants doubted the credibility of the UDF, perceiving it as being more business-oriented and comprising quality for cost. P7-“What *they are mainly trying to do is to cut costs wherever they can. So it is not exactly for the student’s benefit.*”

The students expressed dissatisfaction with the UDF for not disclosing the nutritional value of the food it served, its preparation methods, and the absence of a nutritionist who could provide the necessary guidance. Consequently, they preferred to dine outside the facility. Nevertheless, most respondents were satisfied with the food and services provided by UDF.

***Sensory beliefs about food from UDF***

The participants expressed dissatisfaction with the sensory experience provided by the UDF, indicating that it lacked taste. P15- “The *mess people provide a properly balanced diet, but people opt out of the mess because of the taste. So, if the taste factor is improved, people might stay with the mess and get some nutritious food.”*

Most participants expressed dissatisfaction with the quality of UDF food. They noted that the curries contained excessive oil, the dishes lacked sufficient spices, the chapatis were of substandard quality, and the menu exhibited an imbalance with an overabundance of potatoes and insufficient green vegetables.

## Discussion

The conceptual model developed for this study aligns with the theory of planned behavior (Ajzen, 1991) ^12^ and is grounded in students’ perspectives on UDF food and their food choices. The model represents a comprehensive transition process in food choices as they progress through their stay at the campus. On average, the students stay on campus for two to four years, and changes in food habits were observed. Their behavioral beliefs, subjective norms, and preconceived control beliefs impact their attitude toward UDF and their perceptions of the quality of the food it serves. In the first year, the students must dine at the UDF, after which they begin exploring outside options driven by several factors. This is substantiated by the respondents who expressed that they dined at the UDF during the first two years of their study, after which many of them explored other options. Most respondents expressed dissatisfaction with the taste, which was one of the primary causes for their exploring other avenues even though they served unhealthy food. ^20^ While the food lacked nutrition, it served as a source of relaxation. This finding is in line with that of a previous study. ^21^ It was also found that taste was a significant factor driving transitions in food habits. Furthermore, the study also found that taste as a sensory experience varies from one person to another. ^21^ Thus, taste is unique to each person. Therefore, it is difficult to identify the foods that have higher sensory attributes. Hence, sensory appreciation cannot be a means of predicting acceptance of a certain food item. ^22^

In line with past studies, the current study also found that the factors that impacted food choices were taste, ^23^ ^,^ ^24^ prices, ^16^ time availability, ^15^ ^,^ ^25^ convenience, ^26^ academic stress, ^25^ lack of knowledge about nutrition, ^3^ ^,^ ^27^ ^,^ ^28^ absence of display boards notifying nutrient content of each meal, ^29^ and cheap cost of unhealthy food ^15^ compared to healthy foods. ^8^ Nevertheless, the significance of this study could be justified through its contribution to the existing literature, specifically the concern expressed by respondents regarding the absence of a nutritionist at the UDF to provide guidance and advice about the food served at the facility, as well as their limited knowledge of the food preparation methods employed. Furthermore, the students articulated their apprehensions regarding the scarcity of alternative outlets offering nutritious food options on campus. The factors contributing to deviations from nutritionally optimal food choices remained consistent across all academic years, disciplines, and genders. ^8^ Moreover, the study indicates that the UDF should enhance its services under administrative guidance and foster a nutritious food environment on campus that is well-received by the student population. ^30^ ^,^ ^31^ This recommendation aligns with previous research, which emphasized the role of UDF in “responding to young adults’ food preferences, food service cost-effectiveness, efficiency, and students’ health considerations”. ^32^

The participants expressed their concerns regarding the UDF, which prompted them to explore alternative dietary options. This behavior is grounded in the Theory of Planned Behavior (Ajzen 1991), which posits that feedback is essential for effecting necessary behavioral modifications based on current practices. ^12^ The conceptual model effectively illustrates the transition in food choices. According to this theory, “background factors,” such as age, education, religion, income, general attitude, and emotions, indirectly influence an individual’s intentions and behavior by affecting behavioral, normative, and control beliefs. The data analysis elucidates insights on study program duration as a background factor influencing participants’ intentions and behavior regarding food choices. The study reveals that the respondents demonstrated a positive disposition toward modifying their attitudes and adopting healthy food habits. This finding aligns with other studies that have established intentions as a precursor for accomplishing a particular behavior. Success in accomplishing behavior is not solely influenced by the intention to perform the behavior but also by other control factors, such as requisite opportunities and resources. These requisite opportunities, resources, and intention to perform behavior collectively influence a successful transition towards the desired behaviour. ^23^ This is well-illustrated in the conceptual model in which these controls are in the form of volitional controls and budgetary constraints affecting students’ intentions and behavior.

The study establishes a relationship between a healthy environment and healthy food choices ^33^ in which the environment facilitates the accessibility and consumption of fruits, vegetables and nutrient-rich food. ^34^ Students could be made aware of the need to consume healthy foods and display the nutritive value chart of the foods served in the UDF in the facility itself. Further, affirmative messages can be shared regularly in the form of posters, charts, digital boards flashing PPTs through social media and other messaging platforms, and other outreach activities. ^35^ Similar findings were noted in other studies also. ^36^

The model was conceptualized and developed based on the in-depth understanding achieved through the study rather than the scale of the sample. Specific university students from diverse regions studying at the same university campus were invited to share their food experiences to accomplish this objective. The sample did not aim to represent the student population; instead, it sought to identify the factors that motivated students to modify their food choices throughout their stay at the campus. Our study sample comprised students from varied social backgrounds exposed to different food cultures, intending to understand their food transition trajectories. The model components illustrate food events that occurred throughout participants’ stay and the role of feedback in attitude development. Future research could consider participants from diverse universities, geographical locations, and educational disciplines. Understanding the significance of UDF and its role in a student’s transitional food journey was not exclusively objective-oriented. Still, it emerged through food events or actions experienced by participants. However, the influence of UDF may vary depending on the university’s geographical location. The significance of the study resides in the scarcity of research in India, elucidating the importance of dietary parameters for students pursuing higher education in distant locations from their residences.

## Conclusion

The conceptual model developed to understand food choices focuses on food actions exhibited by individual participants and their overall impact on the formation of an individual’s attitude toward healthy food consumption. The created model provides university decision-makers with valuable information on encouraging students to choose healthy foods, thereby improving their academic performance. Moreover, the UDF must adapt its services to align with the nutritional needs of the students while ensuring that taste is not compromised, or additional costs are not incurred. Past studies have shown that a conducive environment promotes healthy food habits. The universities can ensure an ideal environment by ensuring easy availability and accessibility to healthy foods. ^8^ ^,^ ^34^ The conceptualized model projected in the paper is a first attempt to understand what governs students’ food choices. Further, research can consider diverse participants in diverse settings.

### Limitations

Food habits studies are multidimensional, individualistic, and circumstances-oriented ^1^ for which specific theory or perspective may not be possible. The participants in the present study were students from technical and health science programs representing a small cross-section of the actual student population in the university. As students progress to the final years of their graduation program, they tend to live in off-campus apartments. ^37^ These students were not considered in the study and can be subject to future research. Another limitation of the study was that the interviews were conducted online, and we may have missed the opportunities provided by face-to-face interactions.

## Data Availability

Figshare. DataPerceptions.docx. DOI: https://doi.org/10.6084/m9.figshare.20766088.v1. ^38^ This project contains the following underlying data:
• Data is transcribed from interviews. Data are available under the terms of the Creative Commons Attribution 4.0 International license (CC-BY 4.0).
