# Conceptualizing a model to study university students’ food choices based on the theory of planned behavior

**DOI:** 10.12688/f1000research.123325.5

**Published:** 2026-01-09

**Authors:** Kshama Vishwakarma, Varalakshmi Chandra Sekaran, Vidya Patwardhan, Asha Kamath

**Affiliations:** 1 Associate Professor, Masters in Personal Management, DHMCT, Welcomgroup Graduate School of Hotel Administration, Manipal Academy of Higher Education, Manipal, Karnataka, 576104, India; 2 Associate Professor, Global Public Health Policy and Governance, Prasanna School of Public Health, Manipal Academy of Higher Education, Manipal, Karnataka, 576104, India; 3 Professor & Coordinator, Centre for Hospitality and Tourism Research, Welcomgroup Graduate School of Hotel Administration, Manipal Academy of Higher Education, Manipal, Karnataka, 576104, India; 4 Department of Applied Statistics and Data Science, Prasanna School of Public Health, P Manipal Academy of Higher Education, Manipal, Karnataka, 576104, India

**Keywords:** Food choices, university students, campus, university dining facility (UDF), qualitative research, healthy food, theory of planned behavior, food transition.

## Abstract

**Background:**

Higher education at the university level is essential for advanced learning, enhancing academic knowledge, and precipitating significant life changes. These include lifestyle adjustments, relocation from one’s primary residence, and the acquisition of autonomy in decision-making. Numerous students opt to reside in dormitories/hostels, resulting in notable alterations in dietary habits. Food habits at campus differ substantially from their previous domestic diets, potentially influencing their overall growth and development during their academic tenure.

**Method:**

The study employs the theory of planned behavior to conceptualize a model and understand university students’ food choices, utilizing qualitative research and a phenomenological approach. Snowball sampling selected 26 undergraduate and postgraduate students from technical and health science programs at a private university in Udupi, who were residing on campus for two to four years. Online interviews were audio-recorded with participants’ consent.

**Results:**

Transcribed interviews were coded and categorized to identify themes, which were subsequently conceptualized to develop a model based on the theory of planned behavior. The model illustrated the duration of students’ campus residence, provided insight into their perspectives on food events and consumption at the university dining facility (UDF), influenced by factors such as palatal preferences, cost considerations, temporal constraints (during academic activities), accessibility of nutritious options, academic-related stress, and insufficient nutritional knowledge. Two primary findings emerged: first, recommendation to display nutritional information in the dining facility; second, students exhibited a preference for consuming less healthy food options off-campus due to their lower monetary cost.

**Conclusion:**

The investigation offers valuable insights into the role of UDF in providing nutritionally balanced meals to students, which may contribute to improved health outcomes and enhanced academic performance. This research elucidates the relationship between students’ dietary choices and their subsequent impact on scholastic achievement.

## Introduction

Students pursuing higher education frequently utilize residential facilities provided by their colleges and universities. While residing in a novel environment, students experience significant alterations in their lifestyle, deviating from the established habits maintained in their home environments. ^1^ The transition to university frequently encompasses forming new social connections, exposure to novel experiences, and personal growth. Significantly, this transition coincides with a critical period for establishing enduring habits, ^2^ including a crucial change in dietary preferences. ^1^ An individual’s dietary practices and beliefs during their tertiary education years can significantly influence their habits in adulthood, potentially affecting the probability of developing obesity and associated conditions, such as diabetes and cardiovascular diseases. ^3^ Students opting for more economical dining options in proximity to campus choose to consume meals outside the campus facilities. This behavior, which predisposes them to potential nutrient deficiencies, is corroborated by a study highlighting that insufficient intake of nutritious food ranks among the top six health risk behaviors reported among university students. ^4^

A report by the WHO (2015a) ^5^ states that “healthy diets optimize growth and development over the short and long term. They are characterized by being sufficient and balanced in quantity and quality, containing a diversity of nutrient-dense foods including vegetables, fruits, whole grain cereals, fish, legumes, nuts, modest amounts of animal-source foods, limited in foods and drinks high in saturated and trans fats, added sugars, and salt”. ^5^ A past study ^6^ has reported that students rarely practice WHO guidelines with respect to dietary habits during their stay on campus. A study conducted in Kansas, United States revealed that a significant proportion of university students were classified as obese, and their dietary patterns necessitated modification to incorporate more nutritionally dense foods, particularly those rich in fruits, vegetables, and dietary fiber. ^7^ Furthermore, a low proportion of fruit and vegetable consumption and a high proportion of food containing elevated levels of calories, saturated fats, alcohol, and added sugar were reported among a significant number of university students. ^8^ Many past studies have pointed out that university students do not follow nutritionally rich lifestyles. ^9^ Further, students find choosing food challenging; ^1^ hence, they gradually move away from healthy food habits. ^4^ Studies also show that only 4% of students consume 30% or less of energy from fat and 10% or less from sugar daily. ^7^ Therefore, there is a pressing need to cultivate healthy eating habits among students, particularly considering the increasing prevalence of lifestyle diseases. It is imperative to promote nutrient-rich food behaviors among students, encouraging them to select whole grains over processed foods, incorporate fruits and vegetables into their daily diets, and avoid health-compromising food choices. ^10^

A review study in the Indian context revealed that a substantial percentage of young people have unhealthy eating habits, which negatively impact their overall progress. The issues of failure in achieving academic goals are on a rise, mostly interconnected to unhealthy food habits, and are likely to intensify in the future. This study considers prime unhealthy habits as “under and over-nutrition, common mental issues, non-communicable diseases (NCDs), and stress and anxiety commonly associated with current “nutrition and epidemiological transition.” ^11^

In prior research on nutrition that explored the relationship between attitudes and food consumption, Axelson et al. (1985) conducted a meta-analysis of relevant studies, identifying a minor correlation between attitudes and behavior. A comparable investigation in social psychology during the 1960s precipitated a crisis in attitude research. The late 1960s witnessed the development of numerous structured attitude models, including Ajzen & Fishbein’s (1980) Theory of Reasoned Action and its extension, the Theory of Planned Behaviour (TPB; Ajzen, 1988). These models have been extensively employed in social psychology and have recently been applied to issues concerning food choice. The Theory of Reasoned Action seeks to elucidate behavior that is under an individual’s control, whereas the TPB also endeavors to address non-volitional behaviors, goals, and outcomes that are not entirely within an individual’s control. ^12^ The theory of planned behavior (Ajzen, 1991, 2005, 2012) provides an alternative framework for understanding consumer decision-making processes. The theory of planned behavior (TPB) aims to offer a comprehensive model for analyzing the factors that influence specific consumer behaviors. ^13^ Hence, the present study aims to apply Ajzen’s (1991) theory of planned behavior to understand and conceptualize how students can modify their dietary preferences towards healthier options during their stay at university campuses. The theory of planned behavior (TPB) proposed by Icek Ajzen in 1985 is one of the most widely used models for examining intentions and consumer behavior in various contexts, including food consumption. ^13^ The TPB states that behavior is influenced by intention to participate in that specific behavior (consuming healthy food) and perceived control behavior (PCB), for instance, practicing healthy food consumption at the campus irrespective of the environment. Fishbein & Ajzen’s (1975) theory of reasoned action suggests that an individual’s intentions serve as the most reliable predictor of their behavior. The intention to engage in a particular behavior is posited to be influenced by both an attitudinal component and a normative component. The latter, referred to as the subjective norm, encompasses the individual’s perception of social pressure to perform the behavior, along with their motivation to conform to these expectations (parental influence and university policy). ^14^ ^,^ ^15^ Intentions drive people to take conscious steps to perform a behavior. PCB is the ability of a person to control their behavior, which is in line with Bandurs’ concept of self-efficacy. ^16^ Terry et al. conducted a study of 146 undergraduate students utilizing regression analysis and found that past behavioral experiences (feedback) significantly predicted both intentions and actual behavior. ^13^ In the present qualitative study, participants had to compulsorily subscribe to UDF during their first year of undergraduate program as per university policy. Postgraduate students were not restricted by policy. The rationale for selecting qualitative methodology was that studies have demonstrated that specific societal groups exhibit diverse attitudes, motivations, and behaviors concerning healthier eating habits. ^17^ In-depth interviews ^18^ provide a methodological tool for investigating food choices from an individual perspective and allow for an in-depth study of the problem. The approach to investigating previous behaviors (experiences) involved examining the period during which students engaged in selecting food, commencing from their first year, and analyzing how this process contributed to the development of feedback-initiated repetitive behavior. This study uses a qualitative approach to examine this subject. In contrast, previous studies exclusively employed a quantitative approach to investigate behaviors related to food consumption. ^19^

## Methods

A private university in Udupi was selected for this study because it has four UDFs. The university has outsourced the management of four University Dining Facilities (UDFs) to a catering service company. These facilities provide four meals daily: breakfast (7:00-9:00 a.m.), lunch (12:30-2:00 p.m.), an evening snack (4:30-6:00 p.m.), and dinner (7:30-9:30 p.m.). Meal times may vary depending on the campus location or student class schedules. The menus are designed to accommodate regional variations and are planned according to the diverse Indian cuisines and student preferences. Meals are nutritionally balanced, incorporating proteins, carbohydrates, and dietary fibers. Given the university’s location in South India, the meals emphasize regional and seasonal produce to effectively manage food costs. During the first year of enrollment, students are required to pay an annual advance at the time of admission. From the second year onward, students have the option to make payments in advance on a weekly, monthly, or quarterly basis, according to their preferences. Universities attract students from all over the country with varied food habits. Second, first-year students dine at the UDF for one year. For this study, the duration of one year and consecutive years thereafter were considered integral to developing feedback on UDF food.

Accordingly, undergraduate students in years II to IV and postgraduate students from the university were selected. As Creswell (2018) ^20^ asserted, phenomenological studies describe the common meaning of a phenomenon being studied by all participants. “It seeks to reduce individual experiences to a description of a universal essence”. ^20^ Hence, this study employed a qualitative research method and an interpretive phenomenological approach to analyze the data and gain insights. Snowball sampling was used to recruit the participants. An in-depth interview was used to gather data and provide more subjective views. The researcher had no prior contact or relationship with the participants before conducting the interviews. Students from the engineering college were initially contacted and invited to participate in this study. According to the inclusion criteria, participants had to be residents of the university accommodation, with ages ranging from 18 to 24 years, and pursuing years II through IV of their study programs. First-year undergraduate students were excluded from the study because they were younger than 18 years old. Initially, a subset of participants was selected through purposive sampling, who subsequently recommended additional potential participants from WhatsApp groups. Undergraduate students from years II through IV and postgraduate students from years I and II of their respective study programs were invited to participate via WhatsApp. Participation was voluntary, and verbal consent was obtained before participation. The interviews were conducted online. The interviews lasted 30-40 minutes.

Participants’ involvement was limited to data collection, and transcripts were discussed exclusively within the research team.

An ethics clearance certificate was obtained from the Kasturba Medical College and Kasturba Hospital Institutional Ethics Committee before recruiting participants for the study. The doctoral committee approved an in-depth interview guide for PhD studies, and experts from the fields of nutrition, culinary arts, and qualitative research validated it. A pilot study was conducted to evaluate the interview guide, and necessary modifications were implemented based on these findings. The interview guide consisted of 8 to 10 questions, with additional probes introduced based on the participant’s responses. The questions included: 1. Demographic information (such as program, education stream, age, etc.), 2. An inquiry into their regular dietary habits while residing on campus from the first year to the final year, 3. An exploration of their perspectives on efforts made to adhere to healthy diets, 4. An examination of their food purchasing behavior both on campus and at their residence, 5. Their perspectives on the dietary habits of their peers, 6. Their behavior regarding ready-to-eat food consumption, 7. Their experiences with the university dining facility, and 8. Their perceptions of healthy dietary habits.

Participants provided verbal consent during online interviews conducted via the MS Teams platform, and the interaction was audio-recorded. Online interviews were conducted with twenty-six students from various regions of India residing on campus. Data collection continued until saturation was achieved. Two students were unable to participate because of their commitment to the examination. The research team was present during online interviews with students who were at their places of residence because of the lockdown. The interviews were conducted in English and transcribed.

Data processing was performed using the deduction approach. Codes were identified and categorized into code families. The data were manually coded ^21^ and analyzed through thematic analysis using ATLAS—ti data management software. The primary researcher and an additional research team member performed coding. Subsequently, the research team reviewed these codes. The coded data were grouped according to their relevance to establish categories. This process facilitated the development of themes and sub-themes. The derived themes were discussed with the research team members and consistency between the data and findings was established.

## Results

The demographic profile of the participants revealed that they were pursuing courses in engineering, architecture, medicine, dentistry, pharmacy, and forensic medicine. Their ages ranged from 18 to 24 years, and most of them belonged to Mumbai, Kolkata, Ahmadabad, Karkala, Ghaziabad, Bangalore, and Kochi. Table 1 shows participants’ profiles. All participants met the educational requirements for their respective programs, with undergraduate students in their second to fourth years and master’s students in their first or second years. Proficient in English.

**Table 1.** Participants profile.

Particulars	Number	Frequency
Medicine	13	50%
Technical	13	50%
UG	16	65.5%
PG	10	34%
Male/Female		70%/30%
City of Residence	Kerala, Bangalore, Hyderabad, Indore, Mumbai, Ahmadabad, Karkala, Udupi, Belgaum, Mysore, Coimbatore, Chennai, Kochi	

During data analysis, each participant was assigned a unique code, denoted as P1, P2, P3, and so on. The following section describes each theme that emerged through data analysis using the quotes of participants. The themes included the following components: 1. Individual behavior beliefs, 2. Normative beliefs, 3. Control beliefs, 4. Actual controls, and 5. Feedback. The themes developed through this analysis were aligned with the theory of planned behaviour to conceptualize the model. The conceptualized model is displayed at the end of the theme description.

### Individual behavioral beliefs

Individual behavioral beliefs were developed based on participants’ experiences with respect to their food consumption routines, knowledge about healthy food, views on nutrient requirements, perceptions about the after-effects of adopting unhealthy food choices, and their observations of their peers’ food choices. Individual beliefs influenced the development of attitudes and intentions regarding the adoption of healthy food choices.

### Food consumption

The data analysis revealed that all participants recognized the importance of consuming healthy food and made an effort to do so whenever possible. P10-“ *I eat almonds. I have a box of almonds in my room, so I eat that when I am hungry or studying, like maybe 20 – 30 I eat.*” The data sample also showed that students consciously added fruit and vegetable juices, almonds, boiled eggs or omelets, idli, and dosa (rice-based breakfast items) to their diets. P11, “I *have tried to make sure I take juice or go to the market and buy an apple, grapes or pineapple. When I became conscious about my health, I used to have oats.*”

The data also showed that the UDF served non-vegetarian food items (fish and chicken) as a protein source. Furthermore, they also served vegetable juices, dals (pulses)-based curries, and rice-based items as part of their main meals. P13-“I only tolerate the mess dal because *I need proteins. In the morning, we get upma, bread/butter, dosa, idli, and all of these things. So carbs are there. We mostly obtain fibers when we eat fruit. And if I get a glass of milk, I get my vitamins there.*” Our data confirmed that students understood the importance of consuming nutrient-rich food in maintaining health and achieving academic success. They also had a positive attitude towards UDF and the food they served because it included nuts, fruits, vegetables, and proteins.

### Knowledge about Healthy food and nutrition

The participants demonstrated limited knowledge regarding the daily nutritional requirements obtainable through an appropriate diet, and believed that freshly prepared food represents the optimal approach to managing their nutrient intake.

Data analysis revealed that participants knew the UDF served “balanced and wholesome meals.” P7- *“I prefer fresh food, freshly cooked food”*.

P8-“For *daily nourishment, I prefer the mess (UDF) food. So, I try to eat my meals in the mess (UDF), as much as possible, because they provide quite a wholesome diet*”.

Our investigation into student behavior revealed that participants deliberately incorporated fresh fruits and nuts into their meals, while avoiding food from restaurants, processed foods, ready-to-eat foods, and fried foods. They also expressed the perception that rice is a healthier alternative to noodles. However, the students demonstrated a lack of knowledge regarding the nutritious composition of various food items, which presented a significant barrier to adopting healthier dietary practices.

### Perspectives on food choices made by peers

Peers’ food consumption habits greatly affected individual food choices. Our data confirmed the belief that participants made unhealthy food choices based more on taste than on the actual nutritional value of the food. Data confirmed that peer influence plays a significant role in developing an unhealthy food environment on campus. P3-“In *my friend circle, only 20 to 30% of students are conscious, and about I would say 30 to 40% are at least a little conscious about healthy food.*” Participants shared that maximum student population was indifferent to nutrient-rich food and mostly consumed food based on taste, accessibility, and convenience (quick-service food). P4-“ *They go more for taste; they want more spicy gravies, paneers, or pasta. Something is tastier, and they don’t care if the food is nutritional. It has to be tasty; this is a primary objective.*” Based on participants’ beliefs the student population at campus could be categorized into different groups including one third, inconsistently practiced healthy food habits, followed a certain diet, and exercised regularly, but preferred cheat diets on weekends. Very few individuals practiced strict diets, regularly participated in physical activity, and followed fad diets, yet they were found to consume fewer nutrients than required. P 20-“ *My experience is that people are now becoming health-conscious; some are fitter, and others starve themselves and take fewer nutrients. This generation does not know; they do not know how much the body needs, how much intake is required, how to take it, and they lack this information*”. The data analysis confirmed that there was an unhealthy food behaviour on campus, mainly attributable to a lack of proper knowledge and guidance about healthy food, and partly because many believed it to be part and parcel of campus life. Our data also showed that students involved in sports activities practiced healthy food habits (consuming food from UDF or avoiding fast food) irrespective of their surroundings.

### Reasons for unhealthy food choices

Participants expressed that they preferred to dine at the UDF during their first and second years of college because they served healthy and nutrient-rich food. P11-“There *was this stage when I was into healthy food. But then, exams come, and one month before exams, I had to start studying, so I stopped caring for my diet.”*

The participants reported a deterioration in their dietary habits as they advanced through their college years, attributed to the increasing burden of academic responsibilities. Their lives became more disorganized, resulting in limited time to focus on dietary habits and choices. P9-“ *I rarely sleep. My sleep schedule is … I sleep after 3 or 4 a.m. I eat snacks like peanuts, or I eat noodles or a sandwich, or just have coffee*”. Lifestyle changes are induced by academic stress, time devoted to extracurricular activities, and long working hours, among other factors. P15-“ *Once I started eating dry fruits in the morning; however, with such busy schedules, I just forgot to take them on most days. However, as we got more classes, we started missing more lunch breaks, which got messed. So that is why I had to start eating outside at odd hours.*”

P3-“I *would drink milk daily. But my breakfast pattern has changed a lot since last year because I am so heavily involved in many activities. I don’t have the time to think about food and what I eat.*” Thus, our data confirmed that food habits were significantly influenced by factors such as busy schedules, working irregular hours, skipping meals, the high cost of healthy food, and limited access to healthy food. On the other hand, cheap, unhealthy food was readily available, so students gradually transitioned to adopting unhealthy food habits. P5-“Healthier *options are more expensive and less tasty. So, I think students prefer food items that are both cheap and tasty.”* P3-“The *majority of the food I eat is for relaxation. That’s why I don’t bother about nutrition.”*

Students also followed undisciplined sleeping and waking patterns. As a result, they either missed breakfast or picked up ready-to-eat (RTE) food on their way to their classes or activities. P4-“Students *usually skip breakfast because they wake up late. If they have to rush for class, they will pick up a roll or a packet of biscuits or chips*”. Thus, factors influencing their food choices included academic stress, examination stress, a lack of a disciplined lifestyle, a desire to experience new foods, convenience, financial constraints, easy accessibility of unhealthy foods, an inclination to consume non-vegetarian food, and a lack of accessibility to outlets offering healthy foods compared to unhealthy options.

## Normative beliefs

Participants’ normative beliefs regarding subjective norms that promote healthy food behavior on campus encompassed university policies, parental influence, and environmental factors. These subjective norms were observed to exert a positive influence on students’ attitudes towards the UDF.

### University policy

The participants expressed that the university conducted awareness programs on adopting healthy food habits and promoting a healthy culture. P2-“ *1 am gaining some knowledge about the nutrient contents of various food items. 2. We gained knowledge about the specific nutrients that we benefit from these foods. 3. We need to educate the people (students) about the nutrients we should consume.”*

Students expressed that it would help if the university changed the food offered at the UDF, based on its nutrient quality and taste. This would help develop a positive attitude towards UDF.

P15-“ *Then a little bit of motivation is required from our side only; some motivation is required for us to eat fruits and take juices, etc.*”

Participants also expressed that they were aware of lifestyle diseases such as diabetes and obesity, which are now prevalent among young people and are mostly caused by unhealthy dietary habits. P9-“ *I think it’s the diseases, it’s the health problems that everyone is getting. I think it is quite visible in the young generation. People are getting diabetes, obesity problems, and many hormonal problems also. And I think it’s the eating habits. I think the only solution to this is to have at least the basic nutrition in our food.”* Students also expressed that it would greatly help if the university regularly sent out notifications to motivate students to adopt healthy food habits. P20-“How *many nutrients does the body need? Some people take too much; some people take less. The authorities must inform us that this is a requirement and that this is the way to get it. Nobody knows, and nobody calculates how much intake is required. Maybe making charts and mandatory dining at the mess (UDF) would help. Outside food, we should not consider that this food has this much nutrition; your body needs this. Make posters, charts, and PPTs to create awareness.”* Participants also believed that enriching junk food with nutrients could help achieve two goals simultaneously: enhancing nutrient value without compromising taste. P17-“We *have to increase nutrients in junk food (laugh); students will eat.*” Furthermore, the analysis highlighted the importance of improving the taste and quality of UDF food that would motivate the transition to healthy food.

***Parental influence***

The participants expressed that their parents had a significant influence on their food consumption habits. P9-“At *home, we can obtain nutrition. This is because, when we are in the hostel, not everything is accessible. So, while we were there, if we just had some sugary biscuits, like Milkbikis or something, at home, we had an option to choose from. My dad has turned me into a more nutrition-conscious person.*” Parental influence from childhood to adulthood plays a significant role in inculcating healthy food habits.

P6-“ *Because my mother has made sure I have almonds daily since I went to school, I have almonds in my hostel room. I regularly eat Almonds or pistachios.*”

P4 *-“I mean, there are more different kinds of vegetables at home. There are more kinds of sabzis (vegetables) at home, so we don’t consume chips.”*

***Environmental influence***

Participants further expressed that the university could leverage social media to promote a healthy food behaviour on campus. P9-“Some *socially active programs or something can help. Thus, they should be sufficiently attractive to attract attention. Some innovative messaging is required.”*

They also felt that student clubs could play a more proactive role in this direction through education, awareness, and listing facilities outside the campus, where students could purchase healthy food at reasonable rates. P9 *-“I think when I see a place where we can access food, I think I would try it. I think young people would.*”

Participants conveyed that they were aware of the importance of physical activity and consumption of healthy food.

## Control beliefs

The preconceived notions about food influenced the students’ transitional journey towards healthy food, irrespective of peer pressure and social circle, during their stay on campus.

### Volitional controls

Several respondents were not influenced by their immediate surroundings and consistently adhered to their established dietary practices. P18-“Everyone *is going for junk food, but I think there are still some people who are health conscious.*” Several respondents understood the importance of healthy eating habits in improving academic performance. P15-“We *have full days of classes and need to maintain our energy levels. When one tends to eat healthily, they fall ill less often. So, it is also especially important to maintain good health.”*

Commenting on why some students were indifferent to eating healthy, P3 said, “ *It depended on their interests. If they think their fitness is a priority, they will automatically be conscious.*”

P5-“I *participate in events and competitions; I do ultra-running, like 60 km. So, I trained as specializing in long-distance running. I never skipped vegetables.”*

Participants indicated that sports professionals are more inclined to make healthy dietary choices due to the competitive nature of their pursuits, where success is contingent upon their performance. Their aspiration to achieve victory serves as a catalyst for maintaining disciplined eating habits.

### Sources of food outlets on campus

Students were aware that their university campus offered several food outlets, comprising university dining facilities and other food joints or canteens, that sold both freshly cooked food and ready-to-eat food (RTE), along with confectionery. P15-“When *you are under stress, you tend to binge on eating. You buy whatever you get, the available packet food or whatever you crave.*” Participants also admitted that they depended on other food joints when they could not dine in the UDF. Usually, participants frequently visited retail outlets for late-night snacks, as they were closer to their hostels.

### Time availability

Most participants reported experiencing time constraints due to their demanding schedules. Consequently, they opted to consume readily available food rather than seek nutritionally rich alternatives. P19-“College *timings, how far is college, if we have an immediate class after lunch, then we cannot go out, these factors affect what we eat, and so we end up eating some egg roll, that should happen only once in a while.*”

Post-data analysis showed that students were hard-pressed for time because they were far too focused on their studies, preparing for exams, and late-night studies to improve their performance. Furthermore, all these factors together affect sleeping and waking habits. As a result, they had little time to prioritize their health and healthy eating habits.

### Actual controls

Actual controls include means of accomplishing behavior. Our data analysis showed that the students faced budget constraints and a lack of easy access to healthy food outlets. Actual controls influenced their intention to practice healthy food habits.

### Budget constraints

The students also expressed that the budget was a constraint, so they did not have adequate funds to purchase high-quality, nutrient-rich food. Therefore, they preferred to dine at the UDF during the last week of the month and to explore other food options around the campus for the rest of the month. P12-“When *out of money, I eat over there (UDF).”*

P5-“Healthier *options are more expensive or less tasty. So, I think students focus on taste rather than nutritive value.*”

Our analysis suggests that students perceive nutritious food as financially burdensome, leading them to opt for less healthy alternatives. Additionally, taste emerges as a significant factor influencing the choice of unhealthy food options.

### Accessibility to healthy food

Participants also expressed that unhealthy options were more readily available near the campus than healthy options. P9-“ *If there is shop having healthy snacks nearby the place of residence, I think I would try it.*” *P15-“UDF provides a proper balanced diet, but people opt out of the mess (UDF) because the food is not that tasty.”*

For them, UDF was the only facility offering healthy food within their budget. They opined that if the UDF improved the taste and quality of the food it served, they would transition to a healthier eating habit.

### Feedback on UDF (post-behavior)

The Theory of Planned Behavior (Ajzen, 1991) ^13^ describes “Feedback” as the post-behavior understanding of the actions taken (Behavior). Studying the current problem from the perspective of the theory of planned behavior, it was found that students displayed an intention to choose UDF over other sources to access healthy food based on feedback.

### Nutritional Quality and Credibility of UDF

Examination of the data confirmed that the UDF served nutrition-rich food, a fact confirmed by the respondents. P21- “Because *they have divided nutrition of everything, they are performing their duties well.”* While the UDF offered a balanced diet, the respondents expressed concerns about the small portions of protein-rich food items served, and that the facility did not disclose the nutritional value of the foods that could add to their awareness. P24-“Whatever *they provide in mess (UDF) is nutritive. They provide dals and lentils, which are nutritious. It’s completely fine; the food is nutritious.*”

A few participants doubted the credibility of the UDF, perceiving it as being more business-oriented and comprising quality for cost. P7-“What *they are mainly trying to do is to cut costs wherever they can. So it is not exactly for the student’s benefit.*”

The students expressed dissatisfaction with the UDF for not disclosing the nutritional value of the food it served, its preparation methods, and the absence of a nutritionist who could provide the necessary guidance. Consequently, they preferred to dine outside the facility. Nevertheless, most respondents were satisfied with the food and services provided by UDF.

***Sensory beliefs about food from UDF***

The participants expressed dissatisfaction with the sensory experience provided by the UDF, indicating that it lacked taste. P15- “The *mess people provide a properly balanced diet, but people opt out of the mess because of the taste. So, if the taste factor is improved, people might stay with the mess and get some nutritious food.”*

Most participants expressed dissatisfaction with the quality of UDF food. They noted that the curries contained excessive oil, the dishes lacked sufficient spices, the chapatis were of substandard quality, and the menu exhibited an imbalance with an overabundance of potatoes and insufficient green vegetables.

### Conceptualized Model

**Figure 1.**
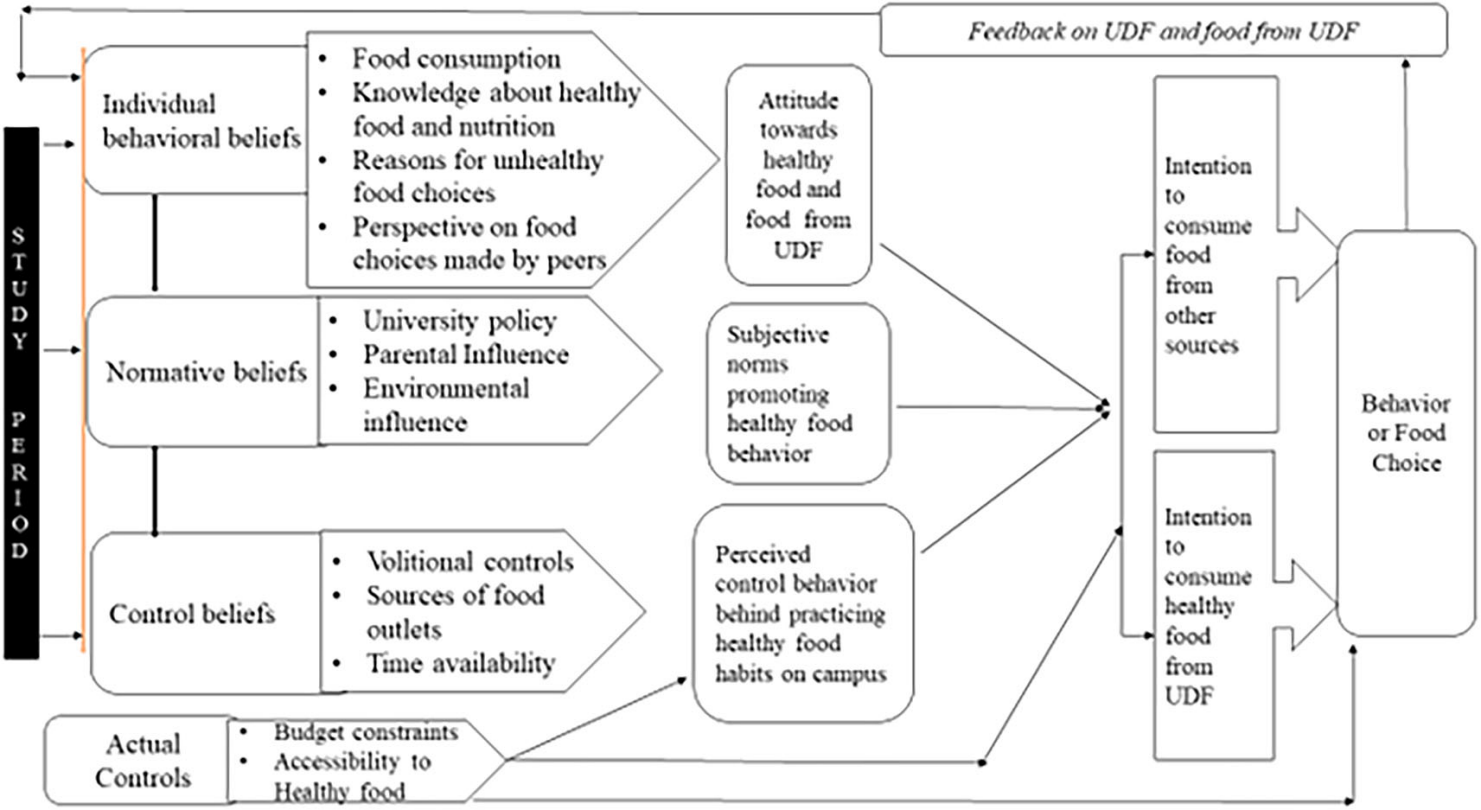
Conceptual model of food choice.

The data analysis results were conceptualized to develop a model that understands university students’ food choices, employing Ajzen’s (1991) ^13^ Theory of Planned Behavior. Figure 1 illustrates the conceptual model of students’ food choices that emerged from the data analysis.

This model suggests that transition begins during the first year on campus and culminates in the development of attitudes towards healthy eating. One of the common sources of food experiences was the university dining facilities (UDF), which students explored through their first to final year. The data elucidated an interdependence with UDF food, and feedback on these food experiences constructed their beliefs about the food provided by these UDFs. Hence, the model illustrates that academic tenure on campus, is one of the background factors which initiates a transformation in students’ dietary habits. Subsequently, this factor influences their intentions to select food from the University Dining Facility (UDF) or to avail food sources of their choice. The study concludes that this cyclical process was evident throughout their campus residency, including the impact of feedback on food behavior (food choice), ultimately leading to informed food selection. This process of food selection could be based on their developed beliefs or attitudes towards healthy eating.

## Discussion

The conceptual model developed for this study aligns with the theory of planned behavior (Ajzen, 1991) ^13^ and is grounded in students’ perspectives on UDF food and their food choices. The model represents a comprehensive transition process in food choices as they progress through their stay at the campus. On average, students stay on campus for two to four years, and changes in their food habits have been observed. Their behavioral beliefs, subjective norms, and preconceived control beliefs impact their attitude toward UDF and their perceptions of the quality of the food it serves. In the first year, the students must dine at the UDF, after which they begin exploring outside options driven by several factors. This is substantiated by the respondents who expressed that they dined at the UDF during the first two years of their study, after which many of them explored other options. Most respondents expressed dissatisfaction with the taste, which was one of the primary causes for their exploring other avenues even though they served unhealthy food. ^22^ While the food lacked nutrition, it served as a source of relaxation. This finding aligns with that of a previous study. ^23^ It was also found that taste was a significant factor driving transitions in food habits. Furthermore, the study also found that taste as a sensory experience varies from one person to another. ^23^ Thus, claiming that taste is unique to each person. Therefore, it is challenging to identify foods with higher sensory attributes. Hence, sensory appreciation cannot be used as a means of predicting the acceptance of a certain food item. ^24^

In line with past studies, the current study also found that the factors that impacted food choices were taste, ^25^ ^,^ ^26^ prices, ^18^ time availability, ^17^ ^,^ ^27^ convenience, ^28^ academic stress, ^27^ lack of knowledge about nutrition, ^3^ ^,^ ^29^ ^,^ ^30^ absence of display boards notifying nutrient content of each meal, ^30^ and the cheap cost of unhealthy food ^17^ compared to healthy foods. ^8^ Nevertheless, the significance of this study could be justified through its contribution to the existing literature, specifically the concern expressed by respondents regarding the absence of a nutritionist at the UDF to provide guidance and advice about the food served at the facility, as well as their limited knowledge of the food preparation methods employed. Furthermore, the students expressed their concerns about the scarcity of alternative outlets offering nutritious food options on campus. The factors contributing to deviations from nutritionally optimal food choices remained consistent across all academic years, disciplines, and genders. ^8^ Moreover, the study indicates that the UDF should enhance its services under administrative guidance and foster a nutritious food environment on campus that is well-received by the student population. ^31^ ^,^ ^32^ This recommendation aligns with previous research, which emphasized the role of UDF in “responding to young adults’ food preferences, food service cost-effectiveness, efficiency, and students’ health considerations”. ^33^

The participants expressed concerns about the UDF, prompting them to explore alternative dietary options. This behavior is grounded in the Theory of Planned Behavior (Ajzen 1991), which posits that feedback is essential for effecting necessary behavioral modifications based on current practices. ^13^ The conceptual model effectively illustrates the transition in food choices. According to this theory, “background factors,” such as age, education, religion, income, general attitude, and emotions, indirectly influence an individual’s intentions and behavior by affecting behavioral, normative, and control beliefs. The data analysis elucidates insights on study program duration as a background factor influencing participants’ intentions and behavior regarding food choices. The study reveals that the respondents demonstrated a positive attitude toward modifying their eating habits and adopting healthier food choices. This finding aligns with other studies that have established intentions as a precursor for accomplishing a particular behavior. Success in accomplishing a behavior is not solely influenced by the intention to perform the behavior but also by other control factors, such as requisite opportunities and resources. These requisite opportunities, resources, and intention to perform behavior collectively influence a successful transition towards the desired behaviour. ^25^ This is well-illustrated in the conceptual model in which these controls are in the form of volitional controls and budgetary constraints affecting students’ intentions and behavior.

The study establishes a relationship between a healthy environment and healthy food choices ^34^ in which the environment facilitates the accessibility and consumption of fruits, vegetables, and nutrient-rich foods. ^35^ Students could be made aware of the need to consume healthy foods and display the nutritive value chart of the foods served in the UDF in the facility itself. Furthermore, affirmative messages can be shared regularly through various channels, including posters, charts, digital boards displaying PowerPoint presentations via social media and other messaging platforms, as well as other outreach activities. ^36^ Similar findings were noted in other studies also. ^37^

The model was conceptualized and developed based on the comprehensive understanding gained through the study, rather than the sample size. University students from various regions studying at the same campus were invited to share their food experiences to achieve this objective. The sample was not intended to represent the entire student population; instead, it aimed to identify the factors that motivate students to change their food choices during their campus tenure. Our study sample included students from diverse social backgrounds, each exposed to different food habits, with the goal of understanding their food transition trajectories. The model components depict food events that occurred throughout the participants’ stay and the role of feedback in shaping attitudes. Future research could include participants from various universities, geographical locations, and academic disciplines. Understanding the significance of UDF and its role in a student’s transitional food journey was not solely objective-oriented; it emerged through food events or actions experienced by participants. However, the influence of UDF may vary depending on the university’s geographical location. The significance of this study lies in the paucity of research in India, highlighting the importance of dietary parameters for students pursuing higher education far from their residences.

## Conclusion

The conceptual model developed to elucidate food choices emphasizes the food-related behaviors exhibited by individual participants and their collective impact on shaping an individual’s attitude toward healthy food consumption. This model offers university decision-makers valuable insights into promoting healthy food choices among students, thereby enhancing their academic performance. Furthermore, the University Dining Facilities (UDF) must adapt their services to meet the nutritional needs of students while ensuring that taste is not compromised and no additional costs are incurred. Previous studies have demonstrated that a conducive environment fosters healthy eating habits. Universities can create an optimal environment by ensuring the easy availability and accessibility of healthy foods. ^8^ ^,^ ^35^ The conceptual model presented in this paper represents an initial effort to comprehend the factors influencing students’ food choices. Future research may consider diverse participants in varied settings.

### Limitations

Food habits studies are multidimensional, individualistic, and circumstances-oriented ^1^ for which specific theory or perspective may not be possible. The participants in the present study were students from technical and health science programs representing a small cross-section of the actual student population in the university. As students progress to the final years of their graduation program, they tend to live in off-campus apartments. ^38^ These students were not considered in the study and can be subject to future research. Another limitation of the study was that the interviews were conducted online, which may have missed opportunities provided by face-to-face interactions.

## Data Availability

Figshare. DataPerceptions.docx. DOI: https://doi.org/10.6084/m9.figshare.20766088.v1. ^39^ This project contains the following underlying data:
• Data is transcribed from interviews. Data are available under the terms of the Creative Commons Attribution 4.0 International license (CC-BY 4.0).
