# Supplementary material for: Conceptualizing a model to study university students’ food choices based on the theory of planned behavior
Source: F1000Res. 2026 Jan 9;11:1192. Originally published 2022 Oct 18. [Version 5] doi: 10.12688/f1000research.123325.5 (PMC12800596; doi:10.12688/f1000research.123325.5)
Supplement: Peer review history

# Reviewer report: Reviewer response for version 5

**DOI:** 10.5256/f1000research.194815.r448952

**Published:** 2026-01-13

**Authors:** Laurel G Lambert

**Affiliations:** 1 Department of Nutrition and Hospitality Management, The University of Mississippi, Mississippi, USA

Suggested changes were applied. 

 New comments: 

 1) Very first sentence has "UDFs". Write out first time, apply acronym and then in the second sentence use acronym.

 2) Delete "Second, first-year students dine at the UDF for one year." Already stated earlier in paragraph.

Is the work clearly and accurately presented and does it cite the current literature?

Partly

If applicable, is the statistical analysis and its interpretation appropriate?

Yes

Are all the source data underlying the results available to ensure full reproducibility?

Partly

Is the study design appropriate and is the work technically sound?

Partly

Are the conclusions drawn adequately supported by the results?

Partly

Are sufficient details of methods and analysis provided to allow replication by others?

No

Reviewer Expertise:

Areas of expertise are child nutrition programs such as school breakfast and lunch and University Dining programs.

I confirm that I have read this submission and believe that I have an appropriate level of expertise to confirm that it is of an acceptable scientific standard.

# Reviewer report: Reviewer response for version 3

**DOI:** 10.5256/f1000research.176562.r383940

**Published:** 2025-05-29

**Authors:** Michelle M. Heer

**Affiliations:** 1 Iowa State University, Ames, USA

I am curious if there were there any relevant changes to the UDFs or food environment because of the lockdown that would fall into the actual controls category?

 A brief summary of the interview methods may be helpful, such as length of time, number of questions, format of questioning such as semi-structured interviews. A copy of the questions in the interview guide may be helpful for further context and replication as the transcript is difficult to sort through to find this information.

 How did you weight the primary findings? Did you utilize frequency of mention to develop the primary findings or just consensus among the research team?

**Perspective on food choices made by peers:** Might this fit into the normative beliefs (subjective norm) category?

 P18-“Everyone *is going for junk food, but I think there are still some people who are health conscious.*” Would this quote fit better under the 'Perspective on food choices made by peers' under the normative beliefs (subjective norm) section, as it appears to pertain to the social expectations of their peers?

 It may be helpful to briefly describe the UDFs in the methods section. What are the typical hours of operation or meal schedule, the price/payment structure, the typical food offerings (possibly utilizing the WHO characteristics of healthy diets).

Is the work clearly and accurately presented and does it cite the current literature?

Partly

If applicable, is the statistical analysis and its interpretation appropriate?

I cannot comment. A qualified statistician is required.

Are all the source data underlying the results available to ensure full reproducibility?

Yes

Is the study design appropriate and is the work technically sound?

Yes

Are the conclusions drawn adequately supported by the results?

Yes

Are sufficient details of methods and analysis provided to allow replication by others?

Partly

Reviewer Expertise:

Food and nutrition, consumer food choice

I confirm that I have read this submission and believe that I have an appropriate level of expertise to confirm that it is of an acceptable scientific standard, however I have significant reservations, as outlined above.

# Response

**Published:** 2025-06-05

**Authors:** Varalakshmi Chandra Sekaran

**Affiliations:** Melaka Manipal Medical College, India

I am curious if there were there any relevant changes to the UDFs or food environment

 because of the lockdown that would fall into the actual controls category?

 During lockdown, the UDF facility was shut down, and whenever students were called on campus, food was packed and served to them. The interviews were done to share their behaviour pre-COVID-19.

 A brief summary of the interview methods may be helpful, such as the length of time,

 the number of questions, and the format of questioning, such as semi-structured interviews. A copy of the questions in the interview guide may be helpful for further context and replication, as the transcript is difficult to sort through to find this information.

 The interview guide question format is added under the method. This publication is part of a PhD study; hence, a copy of the interview guide could not be shared. Hope the information shared is satisfactory.

 How did you weight the primary findings?

 Did you utilize the frequency of mention to develop the primary findings or just the consensus among the research team?

 Yes, the primary findings were based on the frequency of mention, and post-developing findings, the discussions with the research team were undertaken.

 Perspective on food choices made by

 peers: Might this fit into the normative beliefs (subjective norm) category?

 P18-“Everyone is going for junk food, but I think there are still some people who are

 health conscious.” Would this quote fit better under the 'Perspective on food choices made by peers' under the normative beliefs (subjective norm) section, as it appears to pertain to the social expectations of their peers?

 The category was developed based on participants’ perceptions about food choices made by their peers. Hence, the category was under individual beliefs and not under normative beliefs.

 It may be helpful to briefly describe the UDFs in the methods section. What are the

 typical hours of operation or meal schedule, the price/payment structure, the typical food offerings (possibly utilizing the WHO characteristics of healthy diets).

# Reviewer report: Reviewer response for version 3

**DOI:** 10.5256/f1000research.176562.r374087

**Published:** 2025-04-21

**Authors:** Laurel G Lambert

**Affiliations:** 1 Department of Nutrition and Hospitality Management, The University of Mississippi, Mississippi, USA

ROL-lacked depth on TRA.

 Methods:

 The interviews were conducted in advance. In advanced of what? 

 How long were the interviews?

 No description of Normative beliefs in literature or methods. First appears in Results.

 Authors describe the process to developing the interview questions but what were the questions. Which questions represented the different TRA constructs? Seems like the constructs were very loosely applied. For example - Why were students ask on perspective on food choices made by peers, why was it placed in Behavioral Beliefs and why weren't peers included as one of the SN?  

 The process of reviewing transcripts was clear.

 Would be helpful to have table of participant characteristics. 

**Food consumption behavior of peers section**

 It seems that the interviewed participants ate healthy but reported their peers did not? 

 Participants were asked to assess what percentage of their peers practice healthy food habits?

 It is not clear how their data showed that an unhealthy food culture attributed to lack of knowledge and athletes consistently practiced a nutritious diet. How was the food culture measured and were some participants athletes? 

 RTE needs to be written out. It is the first time seen. I am assuming it means = Ready to Eat. 

 There is a great deal of literature on peers influencing eating. Why were peers or teachers not included in subjective norms? 

 In Budget Constraints section - "heavy in their pockets' is an idiom that may need to be explained. Not a familiar use in U.S.

Is the work clearly and accurately presented and does it cite the current literature?

Partly

If applicable, is the statistical analysis and its interpretation appropriate?

Yes

Are all the source data underlying the results available to ensure full reproducibility?

Partly

Is the study design appropriate and is the work technically sound?

Partly

Are the conclusions drawn adequately supported by the results?

Partly

Are sufficient details of methods and analysis provided to allow replication by others?

No

Reviewer Expertise:

University dining services and Theory of Reasoned Action and Planned Behavior.

I confirm that I have read this submission and believe that I have an appropriate level of expertise to confirm that it is of an acceptable scientific standard, however I have significant reservations, as outlined above.

# Response

**Published:** 2025-04-24

**Authors:** Varalakshmi Chandra Sekaran

**Affiliations:** Melaka Manipal Medical College, India

Reviewers comments

 ROL-lacked depth on TRA.

 Response: Added in manuscript.

 Methods:

 The interviews were conducted in advance.

 In advance of what?

 How long were the interviews?

 Response: Made changes in manuscript

 No description of Normative beliefs in

 literature or methods. First appears in

 Results.

 Response: Made changes in the manuscript

 Authors describe the process to developing

 the interview questions but what were the

 questions. Which questions represented the

 different TRA constructs? Seems like the

 constructs were very loosely applied. For

 example -

 Why were students ask on perspective on

 food choices made by peers, why was it

 placed in Behavioral Beliefs and why

 weren't peers included as one of the SN?

 Response: Questions are shared via the DOI with the journal. The question related to peers was asked to find their food environment on campus and their perceptions related to their peers.  During the interview, participants did not share that their peers influenced them to follow healthy food habits. Hence, not included in SN.

 The process of reviewing transcripts was clear.

 Would be helpful to have table of

 participant characteristics.

 Response: Table is added

 Food consumption behavior of peers

 section

 It seems that the interviewed participants ate

 healthy but reported their peers did not?

 Participants were asked to assess what

 percentage of their peers practice healthy

 food habits?

 Response: Made changes to the theme description.

 There is a great deal of literature on peers

 influencing eating. Why were peers or

 teachers not included in subjective norms?

 Response: Data analysis did not report teachers' influence as students pursued undergraduate and master's programs. Peers influence is reported under Food consumption behavior of peers section which was mainly for unhealthy food consumption.

 RTE needs to be written out. It is the first

 time seen. I am assuming it means = Ready

 to Eat.

 Response: Made changes in manuscript.

 How was the food culture measured and

 were some participants athletes?

 Response: Made changes in the manuscript. The use of culture was meant as a food environment. Athletes are students playing sports. Made changes in the manuscript.

 It is not clear how their data showed that an

 unhealthy food culture attributed to lack of Changes are made in the manuscript.

 knowledge and athletes consistently practiced a nutritious diet.

 Response: Changes are made in the manuscript.

# Reviewer report: Reviewer response for version 2

**DOI:** 10.5256/f1000research.161972.r337557

**Published:** 2024-12-30

**Authors:** Yinying Tao

**Affiliations:** 1 Zhejiang University of Science and Technology, Hangzhou, Zhejiang, China

1. The background section is written too simply. It could be written in more detail and cited some of the latest literature as evidence.

 2. Why choose this method? This method is not very innovative, but why is it suitable for the research question of this paper? It is necessary to explain the necessity of using this method and its relevance to this paper.

 3. Are the findings, interpretations and conclusions warranted from the research and data collected?I couldn't find the relevant data processing section in the article.

 4. In the conceptual model, there is no explanation for the selection of influencing factors.

 5. In the discussion section, we can emphasize the significance of this study again.

Is the work clearly and accurately presented and does it cite the current literature?

Partly

If applicable, is the statistical analysis and its interpretation appropriate?

I cannot comment. A qualified statistician is required.

Are all the source data underlying the results available to ensure full reproducibility?

Yes

Is the study design appropriate and is the work technically sound?

Partly

Are the conclusions drawn adequately supported by the results?

Partly

Are sufficient details of methods and analysis provided to allow replication by others?

Partly

Reviewer Expertise:

food design; Tourism management; Heritage protection

I confirm that I have read this submission and believe that I have an appropriate level of expertise to confirm that it is of an acceptable scientific standard, however I have significant reservations, as outlined above.

# Response

**Published:** 2025-01-02

**Authors:** Varalakshmi Chandra Sekaran

**Affiliations:** Melaka Manipal Medical College, India

The background section is written too simply. It could be written in more detail and cited some of the latest literature as evidence.

 Response: The mentioned changes are done in the manuscript. Citation is avoided to abstract guidelines.

 Method:  It is necessary to explain the necessity of using this method and its relevance to this paper.

 Response: The mentioned changes are done in the manuscript

 Discussion: In the discussion section, we can emphasize the significance of this study again.

 Response: The mentioned changes are done in the manuscript

 In the conceptual model, there is no explanation for the selection of influencing factors.

 Response: Wherever possible changes are made in the revised draft

# Reviewer report: Reviewer response for version 1

**DOI:** 10.5256/f1000research.135420.r218221

**Published:** 2023-11-28

**Authors:** Dimitris Skalkos

**Affiliations:** 1 University of Ioannina, Ioannina, Greece

The manuscript is badly written even though the research work can be considered as interesting. 

 The manuscript should be rewritten with the following remarks:

 1. Introduction:

 Recent citations should be use and not old since 2000! As two examples: 

 . FOR REVIEW OF FOOD CHOICE MOTIVES: “Exploring the Impact of COVID‐19 Pandemic on Food Choice Motives: A Systematic Review” D.Skalkos, Z.C. Kalyva, *Sustainability*, 15, 1606, 1-16, https://doi.org/10.3390/su15021606 **2023**

 . FOR STUDENTS' FOOD CHOICES:

 “The impact of the COVID-19 pandemic on college Students’ food choice motives in Greece” **D.Skalkos,** Z.C. Kalyva, I.S. Kosma, *Sustainability*, 15, 9865, 1-11, https://doi.org/10.3390/su15139865 **2023**

 Literature is full of related publications the last 3 years!!

 2. Results:

 The answers of the participants should be presented in a numerical form and table(s) in order for the results to be presented correctly

 The questionnaire itself should be presented in an appendix form

 3. Discussion:

 As with the introduction recent references of related work should be used and not such as 16 (2001), 17 (2008), 27 (1985), 28 (2012) etc.

 My recommendation is: ACCEPTED AFTER MAJOR REVISIONS

Is the work clearly and accurately presented and does it cite the current literature?

No

If applicable, is the statistical analysis and its interpretation appropriate?

No

Are all the source data underlying the results available to ensure full reproducibility?

Partly

Is the study design appropriate and is the work technically sound?

Partly

Are the conclusions drawn adequately supported by the results?

No

Are sufficient details of methods and analysis provided to allow replication by others?

Partly

Reviewer Expertise:

Food business management of innovation

I confirm that I have read this submission and believe that I have an appropriate level of expertise to confirm that it is of an acceptable scientific standard, however I have significant reservations, as outlined above.

# Response

**Published:** 2024-01-29

**Authors:** Varalakshmi Chandra Sekaran

**Affiliations:** Melaka Manipal Medical College, India

Dear Reviewer, thank you for the suggestions to improve our article.

  Introduction:

 Recent citations should be used and not old since 2000!

 The mentioned changes are done in the manuscript and highlighted.

 Results:

 1. The answers of the participants should be presented in a numerical form and table(s) for the results to be presented correctly

 2. The questionnaire itself should be presented in an appendix form

1. A qualitative method was applied for the study hence numerical tables were not added in the paper.
2. The questionnaire is included in the revised transcript. Earlier questionnaire was submitted with a separate DOI

Discussion:

 As with the introduction recent references of related work should be used and not such as 16 (2001), 17 (2008), 27 (1985), 28 (2012), etc.

 Changes are made in revised draft.

# Reviewer report: Reviewer response for version 1

**DOI:** 10.5256/f1000research.135420.r225157

**Published:** 2023-11-28

**Authors:** Rajshri Roy

**Affiliations:** 1 Faculty of Medical and Health Sciences, School of Medical Sciences, The University of Auckland, Auckland, New Zealand; 2 Nutrition and Dietetics, The University of Sydney, Sydney, New South Wales, Australia

**Study Design and Setting: **The choice of a private university in Udupi with four University Dining Facilities as the study setting is rational given its diverse student population and the compulsory dining policy for first-year students. This allows for a broad range of data on food choices over time.

**Participants:** The study focuses on a specific age group (18-24 years) and academic years (II-IV for undergraduates, I-II for postgraduates), which provides a clear scope. However, the exclusion of first-year undergraduates raises questions since their experiences with UDFs in their compulsory dining year could provide valuable insights into initial food choice behaviors.

**Sampling Method:** Using snowball sampling for participant recruitment is common in qualitative studies, particularly when exploring specific behaviors within a defined community. The use of purposive sampling to initiate the snowball sampling process is appropriate, though there is a potential for bias as the initial subjects could influence the sample's homogeneity.

**Data Collection: **The in-depth interview method is suitable for exploring the subjective aspects of food choice. The assurance that the interviewer did not have a prior relationship with the participants helps mitigate bias. However, the study would benefit from clarifying the interview questions or providing an appendix or supplementary file with the interview guide to assess the relevance and comprehensiveness of the questions.

**Data Saturation**: The article should state if the data reached saturation, meaning no new themes were emerging from the interviews.

**Quantitative Data**: If any quantitative measures were used to supplement the qualitative data, these should be presented to strengthen the findings.

**Analytical Rigor**: While thematic analysis is mentioned, more information on how themes were derived and validated would add to the credibility of the results.

 The **discussion and conclusion** are well-aligned with the results, contribute to the academic conversation with new insights, and are presented clearly and accurately with appropriate citations. The authors are candid about the limitations of their work and suggest avenues for future research, which adds to the credibility of the study.

Is the work clearly and accurately presented and does it cite the current literature?

Yes

If applicable, is the statistical analysis and its interpretation appropriate?

Not applicable

Are all the source data underlying the results available to ensure full reproducibility?

Partly

Is the study design appropriate and is the work technically sound?

Partly

Are the conclusions drawn adequately supported by the results?

Yes

Are sufficient details of methods and analysis provided to allow replication by others?

Partly

Reviewer Expertise:

Public Health Nutrition - Obesity - Diet - Food Environment Interventions - Dietary Behaviour Change - Young Adults - Health Promotion - Social Marketing - Implementation Science - Nutrition Assessment - Nutritional Epidemiology - Dietary Patterns - eHealth/mHealth - Qualitative Research - Translational Research​

I confirm that I have read this submission and believe that I have an appropriate level of expertise to confirm that it is of an acceptable scientific standard, however I have significant reservations, as outlined above.

## References

[1] : Description, measurement and evaluation of tertiary-education food environments. *Br J Nutr* .2016;115(9) : 10.1017/S0007114516000568 1598-606 10.1017/S0007114516000568 27245102

# Response

**Published:** 2024-01-29

**Authors:** Varalakshmi Chandra Sekaran

**Affiliations:** Melaka Manipal Medical College, India

Dear Reviewer, thank you for the suggestions to improve our article.

**Study Design and Setting: **The choice of a private university in Udupi with four University Dining Facilities as the study setting is rational given its diverse student population and the compulsory dining policy for first-year students. This allows for a broad range of data on food choices over time.

 Thank you for this observation.

**Participants:** The study focuses on a specific age group (18-24 years) and academic years (II-IV for undergraduates, I-II for postgraduates), which provides a clear scope. However, the exclusion of first-year undergraduates raises questions since their experiences with UDFs in their compulsory dining year could provide valuable insights into initial food choice behaviors.

 First year students were excluded from study-

1. At the time of conduct of the study, newly enrolled students were not yet 18 years of age and did not fit the inclusion criteria.
2. They begin with experiencing food at campus and need few months to develop their feedback.

**Sampling Method:** Using snowball sampling for participant recruitment is common in qualitative studies, particularly when exploring specific behaviors within a defined community. The use of purposive sampling to initiate the snowball sampling process is appropriate, though there is a potential for bias as the initial subjects could influence the sample's homogeneity.

 Thank you for this observation. The study employed purposive sampling initially followed by snowball sampling.

**Data Collection: **The in-depth interview method is suitable for exploring the subjective aspects of food choice. The assurance that the interviewer did not have a prior relationship with the participants helps mitigate bias.

 Thank you for this observation.

 An additional statement is made in the revised manuscript by declaring no relationship was present with participants prior to the study.

**Data Saturation**:

 Data saturation statement is added in the methodology

**Analytical Rigor**: While thematic analysis is mentioned, more information on how themes were derived and validated would add to the credibility of the results.

 This is included in the last paragraph of the methodology.  The development of themes was by an iterative process, with coding followed by categorization into sub-themes and finally into themes.
